# Comprehensive DSRCT multi‐omics analyses unveil CACNA2D2 as a diagnostic hallmark and super‐enhancer‐driven EWSR1::WT1 signature gene

**DOI:** 10.1002/cac2.70015

**Published:** 2025-03-15

**Authors:** Florian Henning Geyer, Alina Ritter, Seneca Kinn‐Gurzo, Tobias Faehling, Jing Li, Armin Jarosch, Carine Ngo, Endrit Vinca, Karim Aljakouch, Azhar Orynbek, Shunya Ohmura, Thomas Kirchner, Roland Imle, Laura Romero‐Pérez, Juan Díaz‐Martín, Stefanie Bertram, Enrique de Álava, Clémence Henon, Sophie Postel‐Vilnay, Ana Banito, Martin Sill, Yvonne Versleijen‐Jonkers, Benjamin Friedrich Berthold Mayer, Martin Ebinger, Monika Sparber‐Sauer, Sabine Stegmaier, Daniel Baumhoer, Wolfgang Hartmann, Jeroen Krijgsveld, David Horst, Olivier Delattre, Patrick Joseph Grohar, Thomas Georg Phillip Grünewald, Florencia Cidre‐Aranaz

**Affiliations:** ^1^ Hopp Children's Cancer Center Heidelberg (KiTZ) Heidelberg Germany; ^2^ National Center for Tumor Diseases (NCT), NCT Heidelberg, a partnership between DKFZ and Heidelberg University Hospital Heidelberg Germany; ^3^ Division of Translational Pediatric Sarcoma Research German Cancer Research Center (DKFZ), German Cancer Consortium (DKTK) Heidelberg Germany; ^4^ Faculty of Medicine Heidelberg University Heidelberg Germany; ^5^ Children's Hospital of Philadelphia Philadelphia Pennsylvania USA; ^6^ Department of Pathology Charité‐University Medicine Berlin, Corporate Member of Freie Universität Berlin and Humboldt‐University of Berlin Berlin Germany; ^7^ German Cancer Consortium (DKTK), Partner Site Berlin, German Cancer Research Centre (DKFZ) Heidelberg Germany; ^8^ Sarcoma Unit, Gustave Roussy Cancer Campus Villejuif France; ^9^ Division of Proteomics of Stem Cells and Cancer German Cancer Research Center (DKFZ), and Heidelberg University Medical Faculty Heidelberg Germany; ^10^ Institute of Pathology, Faculty of Medicine, Ludwig‐Maximilian‐University of Munich Munich Germany; ^11^ Department of Pediatric Oncology, Hematology and Immunology Heidelberg University Hospital Heidelberg Germany; ^12^ Soft‐Tissue Sarcoma Junior Research Group, DKFZ Heidelberg Germany; ^13^ Institute of Biomedicine of Seville (IBiS)/University Hospital Virgen del Rocío/CSIC/University of Seville/CIBERONC Seville Spain; ^14^ Department of Human Anatomy and Embryology, Faculty of Medicine University of Seville Seville Spain; ^15^ Institute of Pathology, University Hospital Essen Essen Germany; ^16^ Department of Normal and Pathological Histology and Cytology, Faculty of Medicine University of Seville Seville Spain; ^17^ Department of Pathology University Hospital Virgen del Rocío Pathology Unit Seville Spain; ^18^ ERC Chromatin Remodeling, DNA Repair, and Epigenetics Laboratory, ARC Team for Fundamental Research, INSERM U981, and Drug Development Department Gustave Roussy Villejuif France; ^19^ University College of London Cancer Institute London UK; ^20^ Division of Pediatric Neurooncology German Cancer Research Center (DKFZ) and German Consortium for Translational Cancer Research (DKTK) Heidelberg Germany; ^21^ Department of Medical Oncology Radboud University Medical Center Nijmegen The Netherlands; ^22^ Department of Pediatric Surgery and Pediatric Urology University Children's Hospital Tübingen Germany; ^23^ Department of Pediatric Hematology and Oncology University Children's Hospital Tuebingen Germany; ^24^ Stuttgart University Hospital gKAöR, Olgahospital, Stuttgart Cancer Center, Center for Child, Adolescent, and Women's Medicine, Pediatrics 5 (Pediatric Oncology, Hematology, Immunology) Stuttgart Germany; ^25^ University of Medicine Tübingen Tübingen Germany; ^26^ Bone Tumor Reference Centre, Institute of Medical Genetics and Pathology, University Hospital Basel, University of Basel, and Basel Research Centre of Child Health Basel Switzerland; ^27^ Gerhard‐Domagk‐Institute of Pathology, University of Muenster Muenster Germany; ^28^ Diversity and Plasticity of Pediatric Tumors, Paris Sciences and Letters University, SIREDO Oncology Centre, Institut Curie Paris France; ^29^ Institute of Pathology, Heidelberg University Hospital Heidelberg Germany

**List of Abbreviations**
CACNA2D2: Calcium voltage‐gated channel auxiliary subunit alpha2delta 2
ChIP‐seq: Chromatin Immunoprecipitation followed by Sequencing
DEG: Differential gene expression
DEP: Differential protein expression
DOX: Doxycycline 
DSRCT: Desmoplastic small round cell tumor
EWSR1: EWS RNA binding protein 1 
fGSEA: Fast gene set enrichment analysis
fp‐ARMS: Fusion‐positive alveolar rhabdomyosarcoma
H3K27ac: Histone H3 lysine 27 acetylation
IQCG: IQ motif containing G 
IRS: Immune Reactive Score
KD: Knock down 
log_2_FC: Log2 fold change 
NES: normalized enrichment score
Padj: Adjusted P‐value 
qPCR: Quantitative polymerase chain reaction
RNA‐seq: RNA‐sequencing
sc: single‐cell
scRNA‐seq: single‐cell RNA‐sequencing
SE: Super enhancer 
shRNA: Short hairpin RNA
ssGSEA: Single sample gene set enrichment analysis
WT1: Wilms tumor protein 

1

Desmoplastic small round cell tumor (DSRCT) is an aggressive cancer that predominantly affects adolescents and young adults, typically developing at sites lined by mesothelium [1, 2]. DSRCT is genetically defined by a chromosomal translocation that fuses the N‐terminus of EWS RNA binding protein 1 (*EWSR1*) to the C‐terminus of Wilms tumor protein (*WT1)*, forming EWSR1::WT1 [3]. This fusion encodes a potent transcription factor and is the only known driver of oncogenic transformation in DSRCT [4]. The lack of a comprehensive understanding of DSRCT biology parallels its dismal survival rate (5%‐20%) [1]. These challenges are exacerbated by the absence of clinical trials, the limited systematic collection and analysis of DSRCT biomaterial [1], and the notable lack of specific diagnostic markers, necessitating resource‐intensive molecular testing for an accurate diagnosis.

Here we first focused on identifying promising candidates for validation as single, fast, and reliable diagnostic DSRCT markers. For this, we performed differential gene expression (DEG) analysis on datasets comprising patient samples from 32 DSRCT and 20 morphological mimics, identifying 23 genes overexpressed in DSRCT (log_2_ fold change (log_2_FC) > 2.5; adjusted *P*‐value (*Padj)* < 0.01; Figure 1A, Supplementary Figure S1A). Secondly, we analyzed EWSR1::WT1 binding sites derived from chromatin immunoprecipitation followed by sequencing (ChIP‐seq) data [5] obtained from the JN‐DSRCT‐1 cell line, identifying 2,065 genomic loci likely regulated by EWSR1::WT1 (Figure 1A). Third, we established JN‐DSRCT‐1 and SK‐DSRCT2 cell lines expressing doxycycline (DOX)‐inducible short hairpin RNA (shRNA)‐mediated EWSR1::WT1 knockdown (KD) (Supplementary Figure S1B). Differential protein expression (DEP) analysis of these cells identified 104 proteins consistently regulated across both cell lines (log_2_FC > 1.0 and *Padj* < 0.01; Figure 1A, Supplementary Table S1). The intersection of these analyses revealed calcium voltage‐gated channel auxiliary subunit alpha2delta 2 (CACNA2D2) and IQ motif containing G (IQCG) as potential DSRCT biomarkers (Figure 1A). *CACNA2D2* was selected for validation due to its significantly higher expression in DSRCTs compared to *IQCG* (*P* < 0.001; Figure 1A). Indeed, DSRCT exhibited the highest expression of *CACNA2D2* among all studied morphological mimics and normal tissues (*P <* 0.001; Supplementary Figures S1C‐D). Further ChIP‐seq data and motif analyses of EWSR1::WT1 binding coordinates and histone marks in JN‐DSRCT‐1 and four DSRCT patient samples [5, 6] suggested a direct regulatory role of EWSR1::WT1 through an enhancer interaction at the *CACNA2D2* locus (Figure 1B). Notably, KD of EWSR1::WT1 in JN‐DSRCT‐1 resulted in a loss of the EWSR1::WT1 signal and Histone H3 lysine 27 acetylation (H3K27ac) enhancer marks at the *CACNA2D2* locus (Figure 1B). Additionally, chromatin interaction data [6] revealed 19 loops connecting the EWSR1::WT1 binding site to the transcription start site of *CACNA2D2*, which were depleted upon KD of *EWSR1::WT1* (Figure 1C). Super enhancer (SE) analysis further demonstrated that the EWSR1::WT1‐bound enhancer exhibited a characteristic SE H3K27ac profile in JN‐DSRCT‐1, which was lost upon EWSR1::WT1 KD (Figure 1D, Supplementary Table S2).

**FIGURE 1:**
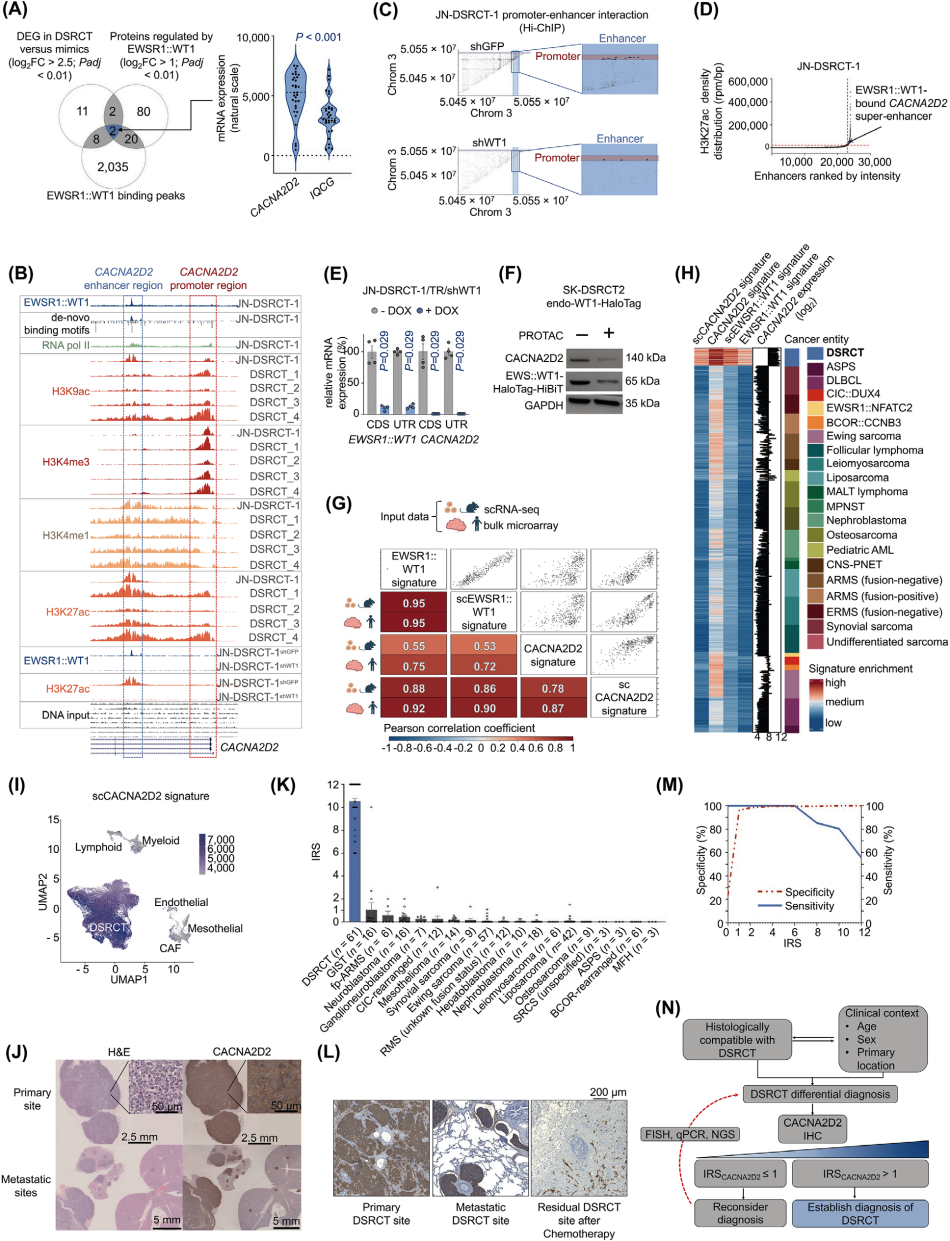
CACNA2D2 is a diagnostic hallmark and super‐enhancer‐driven EWSR1::WT1 signature gene. (A) Left: Venn diagram showing the overlap of genes significantly overexpressed (log_2_FC > 2.5; *Padj* < 0.01) in DSRCT compared to all other tumor entities (as in Supplementary Figure S1A) with genes or proteins potentially regulated by EWSR1::WT1, as determined by ChIP‐seq and Mass spectrometry analyses. Right: Violin plot depicting *CACNA2D2* and *IQCG* mRNA expression in 32 DSRCT patient samples. The black dotted line indicates the median, while blue dotted lines indicate quartiles. Statistical analysis: unpaired two‐sided Mann‐Whitney test. (B) EWSR1::WT1 de novo binding motifs and the epigenetic profile of the *CACNA2D2* locus (chromosome 3:50,506,517‐50,548,707, hg19) from four DSRCT tumor samples (DSRCT_1‐4), JN‐DSRCT‐1 wild‐type, and JN‐DSRCT‐1 cells carrying an shRNA against GFP (control) or EWSR1::WT1. ChIP‐seq profiles from GSE156277 and GSE212977 are depicted for EWSR1::WT1 (blue), RNA polymerase II (green), H3K4me3 (red), H3K4me1 (yellow), H3K27ac (orange), and H3K9ac (salmon). Rectangles highlight the *CACNA2D2* promoter and enhancer regions. (C) Scatter plot showing spatial chromatin interactions between genomic loci in chromosome 3, captured by Hi‐ChIP (GSE212978). Dot density reflects the frequency of interactions. Highlighted dots represent interactions between the *CACNA2D2* promoter and enhancer loci. The *CACNA2D2* promoter region is marked in red, and the enhancer region is marked in blue. (D) Graph depicting H3K27ac signal density at active enhancer sites in the JN‐DSRCT‐1 cell line (GSE212977), ranked by normalized intensity. Red lines indicate the cutoff for super‐enhancer identification (n = 859). (E) Bar plot showing relative mRNA expression levels of *EWSR1::WT1* and *CACNA2D2*, quantified by qRT‐PCR in JN‐DSRCT‐1 cell line expressing a DOX‐inducible shRNA‐mediated KD of EWSR1::WT1 for 96 hours. n = 4 biologically independent experiments. Horizontal bars represent mean expression levels, and whiskers indicate SEM. The number of analyzed samples is given in parentheses. Statistical analysis: unpaired two‐sided Mann‐Whitney test. (F) Western blot using antibodies against CACNA2D2, (EWSR1::)WT1, and GAPDH (loading control) in the SK‐DSRCT2‐endo‐WT1‐HaloTag cell line, which carries a knock‐in of the HaloTag‐HiBiT tag at the endogenous locus of *EWSR1::WT1* C‐terminus. Cells were cultured with 1 µmol/L PROTAC or DMSO as control for 24 hours. (G) x‐y scatter plots illustrating the correlation of ssGSEA enrichment scores between (sc)EWSR1::WT1 and (sc)CACNA2D2 signatures of orthotopically xenografted single DSRCT cells. The numbers and colors in the correlation matrix represent Pearson correlation values for each signature enrichment in single cells from orthotopically xenografted DSRCT (top) or 32 bulk DSRCT patient samples (bottom). (H) Heatmap depicting ssGSEA signature enrichment scores for (sc)EWSR1::WT1 and (sc)CACNA2D2 signatures, along with log_2_ expression values for *CACNA2D2* across 32 DSRCT patient samples and 20 additional cancer entities relevant for DSRCT differential diagnosis. (I) UMAP plot of scRNA‐seq analysis of merged and integrated data from eleven samples, comprising tumor‐derived and normal cells from four DSRCT patients (GSE263523), using the scCACNA2D2 signature. The color gradient indicates the ssGSEA score for scCACNA2D2 signature enrichment. (J) Representative histological images of CACNA2D2 IHC in a DSRCT murine orthotopic xenograft from a primary site and micrometastases in inner organs (muscle, liver, and kidney). DAB (brown chromogen) was used. (K) Bar plot showing individual IRS scores for CACNA2D2 staining in DSRCT and other morphological mimics. The number of analyzed samples is given in parentheses. Bars represent mean IRS values, and whiskers indicate SEM. DSRCT samples are highlighted in blue. Statistical analysis: unpaired two‐sided Mann‐Whitney test. (L) Left: Representative histological image of CACNA2D2 IHC in DSRCT primary tumor tissue. Middle: Representative histological image of CACNA2D2 IHC in a lung metastasis of DSRCT. Right: Representative histological image of CACNA2D2 IHC in a tumor sample with residual DSRCT cells after treatment. DAB (brown chromogen) was used. (M) Graph showing sensitivity (blue solid line) and specificity (red dashed line) of CACNA2D2 IHC, both expressed as percentages. (N) Diagram illustrating the proposed workflow for establishing a robust diagnosis of DSRCT. Abbreviations: AML, acute myeloid leukaemia; ARMS, alveolar rhabdomyosarcoma; ASPS, alveolar soft part sarcoma; BCOR::CCNB3, BCOR::CCNB3 rearranged sarcomas; CACNA2D2, Calcium voltage‐gated channel auxiliary subunit alpha2delta 2 gene; CAFs, cancer‐associated fibroblasts; CDS, coding sequence; ChIP‐seq, chromatin immunoprecipitation followed by sequencing; Chrom, chromosome; CIC::DUX4, CIC::DUX4 rearranged sarcomas; CNS‐PNET, primitive neuroectodermal tumor arising in the central nervous system; DAB, 3,3'‐diaminobenzidine; DEG, differential gene expression; DLBCL, diffuse large B‐cell lymphoma; DMSO, dimethyl sulfoxide; DOX, doxycycline; DSRCT, Desmoplastic small round cell tumor; DSRCT_1 – 4, DSRCT patient sample data; ERMS, embryonal rhabdomyosarcoma; EWS::WT1‐HaloTag‐HiBiT, EWSR1::WT1 protein fused to HaloTag‐HiBiT protein; EWSR1::NFATC2, EWSR1::NFATC2 rearranged sarcomas; EWSR1::WT1, fusion protein of EWSR1 and WT1 proteins; FISH, Fluorescent in situ hybridization; GAPDH, Glyceraldehyde‐3‐phosphate dehydrogenase; GFP, Green fluorescent protein; GIST, gastrointestinal stromal tumor; H&E, hematoxylin and Eosin; H3K27ac, histone H3 lysine 27 acetylation; H3K4me1, histone H3 lysine 4 aminomethylation; H3K4me3, histone H3 lysine 4 trimethylation; H3K9ac, histone H3 lysine 9 acetylation; hg19, human genome version 19; Hi‐ChIP, high‐throughput chromatin immunoprecipitation followed by sequencing; IHC, immunohistochemistry; IQCG, IQ motif containing G gene; IRS, immune reactive score; JN‐DSRCT‐1/TR/shWT1, JN‐DSRCT‐1 cell line stably expressing a doxycycline inducible shRNA expression cassette targeting EWSR1::WT1 mRNA; kDa, kilodalton; log2FC, log2 fold change; MALT, mucosa‐associated lymphoid tissue lymphoma; MFH, malignant fibrous histiocytoma; MPNST, malignant peripheral nerve sheath tumor; NGS, next generation sequencing; Padj, adjusted P‐value; PROTAC, proteolysis targeting chimera; qRT‐PCR, quantitative real‐time polymerase chain reaction; RMS, rhabdomyosarcoma; RNA pol II, RNA polymerase II; rpm/bp, reads er million per base pair; scCACNA2D2, single‐cell data derived CACNA2D2 signature; scEWSR1::WT1, single‐cell data derived EWSR1::WT1 signature; scRNA‐seq, single‐cell RNA‐sequencing; SEM, standard error of the mean; shGFP, shRNA targeting GFP mRNA; shRNA, short hairpin RNA; shWT1, shRNA targeting EWSR1::WT1 mRNA; SK‐DSRCT2 endo‐WT1‐HaloTag, SK‐DSRCT2 cell line expressing endogeneous CACNA2D2 protein fused to HaloTag protein; SRCSs, small round cell sarcomas; ssGSEA, single sample gene set enrichment analysis; UMAP, uniform manifold approximation and projection; UTR, untranslated region.

Post‐transcriptional and post‐translational KD of EWSR1::WT1 in three DSRCT cell line models expressing different EWSR1::WT1 isoforms (Supplementary Figure S2A) resulted in a significant reduction in CACNA2D2 expression (Figures 1E–F, Supplementary Figure S1B, Supplementary Figures S2B–F). Additionally, ChIP‐seq data derived from MeT‐5A mesothelial cells [6] – the potential cell of origin of DSRCT [7, 8] – ectopically expressing different EWSR1::WT1 isoforms (‐KTS, +KTS, or ‐KTS/+KTS) suggested direct regulation, as evidenced by the co‐occurrence of H3K27ac signals and signals for V5‐ or HA‐tagged EWSR1::WT1 isoforms at the *CACNA2D2* enhancer region (Supplementary Figure S2G). Notably, MeT‐5A cells transfected with a control vector showed no substantial signal at this locus (Supplementary Figure S2G). Publicly available RNA‐sequencing (RNA‐seq) data from MeT‐5A cells [6] expressing different EWSR1::WT1 isoforms showed that *CACNA2D2* was differentially expressed in the presence of EWSR1::WT1 (4.1 ≤ log_2_FC ≤ 5.9, *Padj* < 0.001) (Supplementary Figure S2H). Finally, quantitative polymerase chain reaction (qPCR) analysis of MeT‐5A cells stably expressing a DOX‐inducible ectopic EWSR1::WT1 expression cassette confirmed that upon EWSR1::WT1 induction, *CACNA2D2* was significantly and highly overexpressed (Supplementary Figure S2I). Taken together, these results emphasize that EWSR1::WT1 is sufficient to drive *CACNA2D2* expression. SE analysis of MeT‐5A‐derived data strikingly showed that the *CACNA2D2* enhancer bound by EWSR1::WT1 became a SE upon ectopic expression of EWSR1::WT1^− KTS + KTS^ (Supplementary Figure S2J).

To explore whether *CACNA2D2* could serve as a surrogate indicator of oncogenic *EWSR1::WT1* transformation, we defined a *CACNA2D2* gene set and gene signature by performing a correlation analysis of gene expression data from 32 DSRCT patient samples (Supplementary Figure S3A, Supplementary Tables S3‐S4). Next, an EWSR1::WT1 signature was computed by performing a combined DEG analysis of newly generated in vivo and in vitro [4] material derived from three DSRCT cell lines upon *EWSR1::WT1* KD (Supplementary Figure S3A, Supplementary Table S4). Notably, fast gene set enrichment analysis (fGSEA) of the *CACNA2D2* gene set demonstrated a highly significant (*Padj* < 0.001) and strong positive enrichment for the EWSR1::WT1 signature (normalized enrichment score, NES_EWSR1::WT1_ = 3.6). Moreover, single sample gene set enrichment analysis (ssGSEA) of expression data from 32 DSRCT patient samples confirmed that the EWSR1::WT1 signature significantly correlated with that of CACNA2D2 (*r* = 0.75), highlighting a transcriptional interconnection between *CACNA2D2* and *EWSR1::WT1 in situ* (Figure 1G). These observations were further supported by single‐cell (sc)‐derived signatures from orthotopically‐generated tumors using two DSRCT cell lines with DOX‐inducible KD of EWSR1::WT1 at primary (*n* = 221) and metastatic (*n* = 221) locations (Figure 1G, Supplementary Table S4). Indeed, ssGSEA of our single‐cell data showed highly significant correlation between the NES of our generated EWSR1::WT1 and CACNA2D2 signatures (Figure 1G), regardless of tumor location, implying that CACNA2D2‐associated genes are also characteristic features of metastasized DSRCT cells (Supplementary Figure S3B).

To delineate the specificity of the interaction between *CACNA2D2* and *EWSR1::WT1* in DSRCT, we performed ssGSEA using our EWSR1::WT1 and CACNA2D2 signatures on expression data from 20 DSRCT morphological mimics (Figure 1H). Here, non‐DSRCT cancer entities showed significantly lower NES and correlation strength for all signatures compared to DSRCT (Supplementary Figures S3C‐D). These results further emphasized the high specificity of the CACNA2D2 and EWSR1::WT1 interplay in DSRCT. Moreover, both bulk‐ and sc‐derived CACNA2D2 signatures precisely distinguished DSRCT cell clusters from non‐tumor cells in single‐cell RNA‐sequencing (scRNA‐seq) data from four DSRCT patients (*n* = 11 samples) [9] (Figure 1I, Supplementary Figure S3E). Concordantly, all predicted normal cell types within these tumors exhibited low enrichment of both CACNA2D2 signatures (Supplementary Figures S3F‐G).

Further, dimensional reduction of *CACNA2D2*‐associated CpG sites in 24 DSRCT patient samples, compared with 192 samples from 13 morphological mimics [10] revealed distinct clustering of all DSRCT samples, which was unique to *CACNA2D2* compared to other described EWSR1::WT1‐regulated genes or *IQCG* (Figure 1A, Supplementary Figures S4A‐B). Additionally, these *CACNA2D2*‐associated CpG sites exhibited significant (*P* < 0.001) and specific hypomethylation in DSRCT patient samples, collectively suggesting that the *CACNA2D2*‐associated methylation signature is a distinct and specific feature of DSRCT (Supplementary Figure S4C).

To assess the diagnostic utility of CACNA2D2, we optimized a staining protocol for DSRCT cell line xenografts, achieving consistent and robust membranous or cytoplasmatic staining, even uncovering micrometastases (Figure 1J, Supplementary Figure S4D).

Finally, we assembled the largest collection of fresh‐frozen and paraffin‐embedded DSRCT patient samples analyzed to date (*n* = 61), comprising primary, metastatic, and post‐treatment samples, and supplemented it with 249 patient samples from 18 different DSRCT morphological mimics (Supplementary Table S5). CACNA2D2 immunoreactivity was evaluated using a modified Immune Reactive Score (IRS) (Supplementary Material and Methods). Excitingly, DSRCT tumor sections exhibited the highest IRS for CACNA2D2 (IRS_mean_ = 10.5, 6 ≤ IRS_DSRCT_ ≤ 12, *P* < 0.001) (Supplementary Figure S4E‐F), with specificity reaching 98% when applying a cutoff of IRS > 1 (Figure 1K‐M, Supplementary Figure S4E). Indeed, even samples derived from CIC‐ and BCOR‐rearranged sarcomas, as well as fusion‐positive alveolar rhabdomyosarcomas, showed negligible mean protein expression compared to DSRCT (IRS_CIC_ = 0.21, IRS_BCOR_ = 0, IRS_fp‐ARMS_ = 0.56). Furthermore, 100% sensitivity was achieved when applying an IRS cutoff of ≤ 6, implying that DSRCT samples consistently displayed strong staining for CACNA2D2 (Figure 1M). Thus, we recommend a single CACNA2D2 staining for clinically and histologically compatible DSRCT differential diagnosis. If IRS_CACNA2D2_ ≤ 1, the diagnosis should be reconsidered or re‐evaluated using molecular diagnostic procedures (such as fluorescence in situ hybridization, qRT‐PCR, or next‐generation sequencing), if available (Figure 1N). Conversely, if IRS_CACNA2D2_ > 1, a diagnosis of DSRCT may be established. Also, CACNA2D2 staining may be used to rule out DSRCT within the broad spectrum of small‐round‐blue‐cell tumors, potentially offering extensive diagnostic utility.

Finally, the high, specific, and homogenous membranous expression of CACNA2D2 in DSRCT, combined with the highly specific antibody described here, makes CACNA2D2 an ideal candidate for targeted therapeutic approaches, including drug delivery using antibody‐drug conjugates or CAR‐T cell therapy. Future studies should investigate the precise role of CACNA2D2 in DSRCT biology, with a focus on its potential contributions in tumor cell fitness, differentiation, and tumorigenic potential.

In conclusion, here we developed an extensive toolset for DSRCT research (Supplementary Figure S4G), a validated blueprint for how such resources could be harnessed in other cancer entities, and identified CACNA2D2 as a singular, powerful DSRCT biomarker.

## AUTHOR CONTRIBUTIONS

Florian Henning Geyer, Florencia Cidre‐Aranaz, and Thomas Georg Phillip Grünewald conceived the study. Florian Henning Geyer and Florencia Cidre‐Aranaz wrote the paper and drafted all figures and tables. Florian Henning Geyer carried out all in vitro and in vivo experiments and performed all bioinformatic and statistical analyses. Florian Henning Geyer, Alina Ritter, and Thomas Georg Phillip Grünewald performed immunohistochemical evaluation and scoring of tumor samples and TMAs. Florencia Cidre‐Aranaz, Roland Imle, and Ana Banito performed and/or coordinated in vivo experiments. Olivier Delattre provided microarray expression data. Seneca Kinn‐Gurzo performed in vitro experiments on BER cell lines. Tobias Faehling and Clémence Henon performed single‐cell bioinformatic analyses. Karim Aljakouch and Azhar Orynbek performed MassSpec and analyzed MassSpec data. Alina Ritter, Jing Li, Endrit Vinca, Laura Romero‐Perez, Martin Sill, and Shunya Ohmura contributed to experimental procedures. Wolfgang Hartmann and Benjamin Friedrich Berthold Mayer provided clinical and/or histological guidance. Enrique De Álava, Juan Díaz‐Martín, Stefanie Bertram, Sophie Postel‐Vilnay, Martin Ebinger, Monika Sparber‐Sauer, Daniel Baumhoer, Carine Ngo, David Horst, Yvonne Versleijen‐Jonkers, Armin Jarosch, Sabine Stegmaier, and Thomas Kirchner provided clinical samples. Patrick Joseph Grohar, Thomas Georg Phillip Grünewald, and Jeroen Krijgsveld provided laboratory infrastructure. Florencia Cidre‐Aranaz and Thomas Georg Phillip Grünewald supervised the study and data analysis. All authors read and approved the final manuscript.

## CONFLICT OF INTEREST STATEMENT

The authors declare no competing interests.

## FUNDING INFORMATION

The laboratory of Thomas Georg Phillip Grünewald is supported by grants from the Matthias‐Lackas Foundation, the Dr. Leopold und Carmen Ellinger Foundation, the European Research Council (ERC CoG 2023 #101122595), the Deutsche Forschungsgemeinschaft (DFG 458891500), the German Cancer Aid (DKH‐70112257, DKH‐7011411, DKH‐70114278, DKH‐70115315), the Dr. Rolf M. Schwiete foundation, the SMARCB1 association, the Ministry of Education and Research (BMBF; SMART‐CARE and HEROES‐AYA), and the Barbara and Wilfried Mohr foundation. The research team of Florencia Cidre‐Aranaz was supported by the German Cancer Aid (DHK‐70114111), and the Dr. Rolf M. Schwiete Stiftung (2020‐028 and 2022‐31). In addition, this work was delivered as part of the PROTECT team supported by the Cancer Grand Challenges partnership funded by Cancer Research UK, the National Cancer Institute, the Scientific Foundation of the Spanish Association Against Cancer And KiKa (Children Cancer Free Foundation). Florian Henning Geyer, Tobias Faehling, Endrit Vinca, and Alina Ritter were supported by the German Academic Scholarship Foundation. In addition, Endrit Vinca was supported by scholarships from the Heinrich F.C. Behr foundation and the Rudolf and Brigitte Zenner foundation, Tobias Faehling by the Heinrich F.C. Behr foundation, and Florian Henning Geyer and Alina Ritter are supported by the German Cancer Aid through the ‘Mildred‐Scheel‐Doctoral Program’ (DKH‐70114866). This project is co‐funded by the European Union (ERC, CANCER‐HARAKIRI, 101122595). All views and opinions expressed are however those of the authors only and do not necessarily reflect those of the European Union or the European Research Council. Neither the European Union nor the granting authority can be held responsible for them.

## ETHICS APPROVAL AND CONSENT TO PARTICIPATE

In vivo experiments were approved by the government of North Baden and conducted in accordance with ARRIVE guidelines and recommendations of the European Community (86/609/EEC) and UKCCCR (guidelines for the welfare and use of animals in cancer research). Open slides or tissue‐microarrays from human formalin‐fixed, paraffin‐embedded or cryopreserved tissue samples were retrieved from the archives of the Institute of Pathology of the LMU Munich, the Charité Berlin, The Biobank of the Hospital Universitario Virgen del Rocío of Seville, the Hospital Gustave Roussy (Villejuif), the Bone Tumor Reference Center at the University of Basel, the University of Essen, the Cooperative Weichteilsarkom Studiengruppe (CWS) study center, the Klinikum Stuttgart (ethics committee from the Medical Faculty of the Eberhard‐Karls University and University Hospital of Tübingen, approval no. 207/2022BO2), the Radboud University Medical Center, the Pathology Institute of the LMU Munich (approval no. 550‐16 UE), and the University of Heidelberg (approval no. S‐211/2021).

## Supporting information



Supporting information

Supporting information

## Data Availability

The microarray data are deposited at the National Center for Biotechnology Information (NCBI) GEO database with accession codes GSE273438 and GSE273441. All proteomic data is deposited at the PRoteomics IDEntifications database with accession code PXD053786. All other data supporting the findings of this study are available within the article and its supplementary information files, or from the corresponding author upon reasonable request.
