# Supplementary material for: Comprehensive DSRCT multi‐omics analyses unveil CACNA2D2 as a diagnostic hallmark and super‐enhancer‐driven EWSR1::WT1 signature gene
Source: Cancer Commun (Lond). 2025 Mar 15;45(6):702–8. doi: 10.1002/cac2.70015 (PMC12187571; doi:10.1002/cac2.70015)
Supplement: Supplementary file 2 — Supporting information [file CAC2-45-702-s001.docx]

**Supplementary Materials**

**Comprehensive DSRCT multi-omics analyses unveil CACNA2D2 as a diagnostic hallmark and super-enhancer-driven EWSR1::WT1 signature gene**

Florian Henning Geyer^1,2,3,4^, Alina Ritter^1,2,3,4^, Seneca Kinn-Gurzo^5^, Tobias Faehling^1,2,3,4^, Jing Li^1,2,3^, Armin Jarosch^6,7^, Carine Ngo^8^, Endrit Vinca^1,2,3^, Karim Aljakouch^9^, Azhar Orynbek^9^, Shunya Ohmura^1,2,3^, Thomas Kirchner^10^, Roland Imle^1,2,11,12^, Laura Romero-Pérez^13,14^, Juan Díaz-Martín^13^, Stefanie Bertram^15^, Enrique de Álava^13,16,17^, Clémence Henon^18^, Sophie Postel-Vilnay^18,19^, Ana Banito^1,2,12^, Martin Sill^1,2,20^, Yvonne Versleijen-Jonkers^21^, Benjamin Friedrich Berthold Mayer^22^, Martin Ebinger^23^, Monika Sparber-Sauer^24,25^, Sabine Stegmaier^24^, Daniel Baumhoer^26^, Wolfgang Hartmann^27^, Jeroen Krijgsveld^9^, David Horst^6,7^, Olivier Delattre^28^, Patrick Joseph. Grohar^5^, Thomas Georg Phillip Grünewald^1,2,3,29^, Florencia Cidre-Aranaz^1,2,3,*^

^1^Hopp Children’s Cancer Center Heidelberg (KiTZ), Heidelberg, Germany.

^2^National Center for Tumor Diseases (NCT), NCT Heidelberg, a partnership between DKFZ and Heidelberg University Hospital, Heidelberg, Germany.

^3^Division of Translational Pediatric Sarcoma Research, German Cancer Research Center (DKFZ), German Cancer Consortium (DKTK), Heidelberg, Germany.

^4^Faculty of Medicine, Heidelberg University, Heidelberg, Germany.

^5^Children’s Hospital of Philadelphia, Philadelphia, PA, United States.

^6^Department of Pathology, Charité-University Medicine Berlin, Corporate Member of Freie Universität Berlin and Humboldt-University of Berlin, Berlin, Germany.

^7^German Cancer Consortium (DKTK), Partner Site Berlin, German Cancer Research Centre (DKFZ), Heidelberg, Germany.
^8^Sarcoma Unit, Gustave Roussy Cancer Campus, Villejuif, France.

^9^Division of Proteomics of Stem Cells and Cancer, German Cancer Research Center (DKFZ), and Heidelberg University Medical Faculty, Heidelberg, Germany.

^10^Institute of Pathology, Faculty of Medicine, Ludwig-Maximilian-University of Munich, Munich, Germany.

^11^Department of Pediatric Oncology, Hematology and Immunology, Heidelberg University Hospital, Heidelberg, Germany.

^12^Soft-Tissue Sarcoma Junior Research Group, DKFZ, Heidelberg, Germany.

^13^Institute of Biomedicine of Seville (IBiS)/University Hospital Virgen del Rocío/CSIC/University of Seville/CIBERONC, Seville, Spain.

^14^Department of Human Anatomy and Embryology, Faculty of Medicine, University of Seville, Seville, Spain.

^15^Institute of Pathology, University Hospital Essen, Essen, Germany.

^16^Department of Normal and Pathological Histology and Cytology, Faculty of Medicine, University of Seville, Seville, Spain.

^17^Department of Pathology, University Hospital Virgen del Rocío, Seville, Spain.
Pathology Unit, Seville, Spain.

^18^ERC Chromatin Remodeling, DNA Repair, and Epigenetics Laboratory, ARC Team for Fundamental Research, INSERM U981, and Drug Development Department, Gustave Roussy, Villejuif, France.

^19^University College of London Cancer Institute, London, United Kingdom.

^20^Division of Pediatric Neurooncology, German Cancer Research Center (DKFZ) and German Consortium for Translational Cancer Research (DKTK), Heidelberg, Germany.

^21^Department of Medical Oncology, Radboud University Medical Center, Nijmegen, The Netherlands.

^22^Department of Pediatric Surgery and Pediatric Urology, University Children's Hospital, Tübingen, Germany.

^23^Department of Pediatric Hematology and Oncology, University Children's Hospital, Tuebingen, Germany.

^24^Stuttgart University Hospital gKAöR, Olgahospital, Stuttgart Cancer Center, Center for Child, Adolescent, and Women's Medicine, Pediatrics 5 (Pediatric Oncology, Hematology, Immunology), Stuttgart, Germany.

^25^University of Medicine Tübingen, Tübingen, Germany.

^26^Bone Tumor Reference Centre, Institute of Medical Genetics and Pathology, University Hospital Basel, University of Basel, and Basel Research Centre of Child Health, Basel, Switzerland.

^27^Gerhard-Domagk-Institute of Pathology, University of Muenster, Muenster, Germany.

^28^Diversity and Plasticity of Pediatric Tumors, Paris Sciences and Letters University, SIREDO Oncology Centre, Institut Curie, Paris, France.

^29^Institute of Pathology, Heidelberg University Hospital, Heidelberg, Germany.

## ^*^Corresponding author:

Florencia Cidre-Aranaz; Division of Translational Pediatric Sarcoma Research

German Cancer Research Center (DKFZ) & Hopp Children’s Cancer Center (KiTZ)

Im Neuenheimer Feld 280, 69210 Heidelberg, Germany; Phone: +49-6221-42-3717; Fax: +49-6221-42-3721; Email: [florencia.cidrearanaz@dkfz](mailto:florencia.cidrearanaz@dkfz).de.

## Supplementary Materials and Methods

### Provenience of cell lines and cell culture conditions

Human cell lines were obtained from the following repositories and sources: MeT-5A human mesothelial cells (ATTC number: CRL-9444) and HEK293T (ATTC number: CRL-3216) cells were purchased from the American Type Culture Collection (ATCC, Manassas, VA, USA). The human DSRCT cell line JN-DSRCT-1 was provided by Dr. Mikiko Aoki (Fukuoka University, Fukuoka, Japan), SK-DSRCT2 was kindly provided by Dr. Marc Ladanyi (Memorial Sloan Kettering Cancer Center, New York, NY, USA), and BER cells were obtained from the Christus Stehlin Foundation for Cancer Research (Houston, TX, USA) [[1](#_ENREF_1)]. All cell lines were cultured in RPMI-1640 medium (Sigma-Aldrich, St Louis, MO, USA) supplemented with 10% tetracycline-free fetal calf serum (FCS; Sigma-Aldrich), 10,000 U/mL penicillin, and 10 mg/mL streptomycin (Sigma-Aldrich) at 37°C in a humidified atmosphere with 5% CO_2_. Cell lines were routinely tested for mycoplasma contamination using nested PCR. Additionally, cell line identity was regularly verified through short tandem repeat profiling or single nucleotide polymorphism profiling.

### Generation of Doxycycline-inducible short hairpin RNA (shRNA) expressing cells

Human DSRCT cell lines JN-DSRCT-1 and SK-DSRCT2 were transduced with lentiviruses encoding the TET-pLKO-puro vector (Plasmid #21915, Addgene, Watertown, MA, USA), which contains a puromycin resistance gene and a doxycycline (DOX)-inducible expression cassette for short hairpin RNAs (shRNAs) targeting *WT1* (shWT1) or a non-targeting control shRNA (shCtrl) [[2](#_ENREF_2)]. DOX-inducible vectors were generated as described previously [[3](#_ENREF_3)], using either a non-targeting shRNA (shCtrl) or two shRNAs against *WT1* as inserts*,* targeting either the coding sequence (shWT1-CDS) or the 3´-UTR (shWT1-UTR) of the *WT1* RNA transcript (**Supplementary Table S6**). Lentiviral particles were generated in HEK293T cells using the psPAX2 (Plasmid #12260, Addgene) and pMD2.G (Plasmid #12259, Addgene) packaging plasmids. Virus-containing supernatant was collected to infect the human DSRCT cell lines. Infected cells were selected with 1 µg/mL puromycin (Sigma-Aldrich). *WT1* KD in DSRCT cells was achieved by adding 1 µg/mL DOX (Sigma-Aldrich) every 72 hours to the cell culture medium. The resulting cell lines were designated as follows: JN-DSRCT-1/TR/shCtrl, JN-DSRCT-1/TR/shWT1-CDS, JN-DSRCT-1/TR/shWT1-UTR, SK-DSRCT2/TR/shCtrl, SK-DSRCT2/TR/shWT1-CDS, and SK-DSRCT2/TR/shWT1-UTR.

### Transient transfection using small interfering RNAs (siRNA)

Two siRNA sequences, each targeting one side of the EWSR1::WT1 fusion breakpoint (**Supplementary Table S6**), were used to selectively suppress EWSR1::WT1 without affecting wildtype EWSR1 or WT1 expression, as previously described [[4](#_ENREF_4)]. Pooled siRNAs or non-targeting siRNA were complexed with Lipofectamine RNAiMax Transfection Reagent (3 μL) (Thermo Scientific, Dreieich, Germany) for 30 minutes at 37 °C. The siRNAs were added to 5 × 10^5^ BER DSRCT cells with culture medium at a final concentration of 2 nmol/L. After incubation for 48 hours at 37 °C, cells were washed with PBS and processed for RNA or protein extraction.

### CRISPR-Cas9 knock-in establishment and PROTAC-mediated degradation

Clustered regulatory interspaced short palindromic repeats (CRISPR)-Cas9 system was used to knock in the HaloTag-HibiT-Tag [[5](#_ENREF_5), [6](#_ENREF_6)] at the endogenous locus of *EWSR1::WT1.* First, custom crRNA targeting the C-terminus of *EWSR1::WT1* was annealed with tracrRNA. Next, Alt-R™ S.p. HiFi Cas9 Nuclease enzyme (Integrated DNA Technologies, Coralville, IA, USA) was added to the annealed sgRNA to form a ribonucleoprotein (RNP) complex. The RNP complex was delivered into 1 × 10^6^ SK-DSRCT2 cells via transient transfection using the SF Cell Line 4D-Nucleofector™ X Kit and the 4D-NucleofectorTM X Unit (Lonza, Basel, Switzerland) with pulse code DS-1337. Isogenic cell clones were obtained through serial dilution, and knock-in was validated via PCR and subsequent Sanger-sequencing. The resulting cell line was designated SK-DSRCT2-endo-WT1-HaloTag. crRNA and PCR primer sequences are provided in **Supplementary Table S6**. To induce post-translational KD of tagged EWSR1::WT1, a PROTAC (Promega Corporation, Madison, WI, USA) targeting the HaloTag was added to the cell culture medium at a concentration of 1 μmol/L for 24 hours. Knockdown efficiency was then assessed with the Nano-Glo^®^ HiBiT Lytic Detection System (Promega) or western blot.

### Generation of DOX-inducible EWSR1::WT1 expressing mesothelial cells

The human mesothelial cell line MeT-5A was transduced with lentiviruses encoding the pTPin overexpression vector, which contains a puromycin resistance gene and a DOX-inducible expression cassette of *EWSR1::WT1^-KTS^* as previously described [[7](#_ENREF_7)]. The DOX-inducible vector was generated by enzymatic digestion with AgeI and NotI restriction enzymes. Total RNA from the SK-DSRCT2 wild-type cell line was reverse-transcribed using SuperScript™ IV Reverse Transcriptase (Thermo Scientific) and used to amplify the *EWSR1::WT1* mRNA sequence. The *EWSR1::WT1* cDNA sequence was amplified using primers with overhangs for AgeI and NotI restriction sites. The PCR product was digested and ligated into the digested pTPin vector. PCR primer sequences are provided in **Supplementary Table S6.** Vectors were amplified in NEB^®^ Stable Competent *E. coli* (New England Biolabs, Ipswich, MA, USA) under 100 μg/mL Ampicillin selection in LB Broth at 30°C and 180 rpm for 18 hours. The integrated transgene was verified by Sanger-sequencing. Lentiviral particles were generated in HEK293T cells using psPAX2 (Plasmid #12260, Addgene) and pMD2.G (Plasmid #12259, Addgene) packaging plasmids. Virus-containing supernatant was collected to infect the mesothelial cell line. Infected cells were selected with 1 µg/mL puromycin. Expression of *EWSR1::WT1* was achieved by adding 1 µg/mL DOX every 72 hours to the culture medium. The resulting cell line was designated MeT-5A-EWSR1::WT1 ^- KTS^.

### RNA extraction, reverse transcription, and quantitative real-time PCR (qRT-PCR)

Total RNA was isolated using the NucleoSpin RNA kit (Macherey-Nagel GmbH & Co. KG, Düren, Germany). 1 µg of total RNA was reverse-transcribed using the High-Capacity cDNA Reverse Transcription Kit (Applied Biosystems, Waltham, MA, USA). qRT-PCR reactions (final volume 15 µl) were performed using SYBR™ Select Master Mix for CFX (Applied Biosystems, Waltham, MA, USA), mixed with diluted cDNA (1:10) and 0.5 µM forward and reverse primers. Reactions were analyzed using CFX Maestro 2.0 software (Bio-Rad Laboratories GmbH, Feldkirchen, Germany). Gene expression values were calculated using the ^ΔΔ^Ct method, with *RPLP0* as the internal housekeeping gene control. Oligonucleotide sequences are provided in **Supplementary Table S6**.

### Analysis of published chromatin immuno-precipitation followed by high-throughput DNA sequencing (ChIP-seq) data and binding motif analysis.

Preprocessed ChIP-seq data from JN-DSRCT-1 cell line were downloaded from the Gene Expression Omnibus data repository with accession codes GSE156277 and GSE212977. ChIP-seq data from four primary DSRCT specimens and the MeT-5A cell line were downloaded from GEO (accession code: GSE212977). The data were visualized in the UCSC genome browser as custom tracks. The samples analyzed in this study are listed in **Supplementary Table S7**. EWSR1::WT1 binding motifs were identified by analyzing previously published genomic positions of EWSR1::WT1 binding sites in Browser Extensible Data (BED) format [[8](#_ENREF_8)] using HOMER’s findMotifsGenome.pl wrapper with hg19 reference genome [[9](#_ENREF_9), [10](#_ENREF_10)]. Find Individual Motif Occurrences (FIMO) analysis was performed using the MEME Suite [[11](#_ENREF_11)]. FIMO was provided with genomic DNA sequences of interest extracted from the UCSC genome browser (hg19) in FASTA format, and the top five de novo and known binding motifs detected in ChIP-seq data of EWSR1::WT1 binding in JN-DSRCT-1 cell line were analyzed. FIMO analysis results were downloaded in General Feature Format (version 3) and uploaded as a custom track to the UCSC genome browser.

### Analysis of published H3K27ac Hi-ChIP data

H3K27ac Hi-ChIP datasets used in this study were downloaded from the Gene Expression Omnibus (GEO) repository (accession code: GSE212978). The published preprocessed data were filtered for high-quality reads (read quality>30). The filtered high-quality reads were plotted in an x-y scatterplot to visualize the spatial interactions between genomic loci captured by the Hi-ChIP experiment, where the x and y coordinates represent the genomic positions of the interacting loci, and the density of points reflects the frequency of interactions. Highlighted areas include the *CACNA2D2* enhancer region in hg19 reference genome (chr3:50,514,500–50,524,800), and the *CACNA2D2* promoter region (chr3:50,538,700–50,542,200).

### Super-enhancer analysis

Raw ChIP-seq data were downloaded as .fastq files from the Sequence Read Archive (SRA) [[12](#_ENREF_12)] and aligned to the hg19 reference genome using the BWA-MEM algorithm [[13](#_ENREF_13)]. SRA run IDs are provided in **Supplementary Table S8**. The resulting .sam files were converted to .bam files, sorted and indexed using samtools [[14](#_ENREF_14)]. H3K27ac .bam files and corresponding DNA input .bam files were used to identify significant ChIP-seq peaks using the macs2 algorithm [[15](#_ENREF_15)]. Resulting .bed file and corresponding sorted and indexed .bam files were used to identify SE using the Rank ordering of SE (ROSE) algorithm [[16](#_ENREF_16), [17](#_ENREF_17)]. Identified enhancers were filtered for known regions on the reference genome and sorted according to their H3K27ac density values.

### Tumor dissociation for scRNA-sequencing

Orthotopically xenografted tumors from SK-DSRCT2 and JN-DSRCT-1 cell lines with EWSR1::WT1 shRNA-KD were processed into single-cell suspensions, as previously described [[18](#_ENREF_18)]. Briefly, tumor sections (≤ 200 mg) were harvested, rapidly cooled in ice-cold PBS, minced with a scalpel, and transferred to gentleMACS C tubes (Miltenyi Biotec, Bergisch-Gladbach, Germany) with 5 mL of ice-cold protease cocktail (cold-active Bacillus licheniformis protease (10 mg/mL), calcium chloride (5 mmol/L), DNase I (125 U/mL) in PBS) for 10 minutes at 4 °C. Samples were processed using a gentleMACS Dissociator (Miltenyi Biotec) at 4 °C using the m_brain_03 program twice. After incubation at 4 °C for 5 minutes, samples were filtered through a 70-µm cell strainer, and 10 mL of ice-cold Hanks' Balanced Salt Solution (HBSS, Corning, Corning, NY, USA) supplemented with 10% tetracycline-free fetal calf serum were added. After centrifugation at 300 × g for 5 minutes at 4 °C, the pelleted cells were resuspended in ice-cold HBSS supplemented with 2% bovine serum albumin. Cells were incubated with Human TruStain FcX™ (Fc Receptor Blocking Solution) (BioLegend, California, CA, USA) and TruStain FcX™ PLUS (anti-mouse CD16/32) antibody (BioLegend) on ice for 5 minutes. APC anti-human CD36 (BioLegend), Brilliant Violet 711™ anti-human CD63 (BioLegend), and FITC anti-mouse CD90.2 (Thy1.2) (BioLegend) antibodies were added at a 1:20 dilution and incubated for 15 minutes on ice. Cells positive for CD36 and CD63 but negative for CD90.2 were defined as DSRCT. 96 cells per tumor sample were sorted into a DNA low-binding 384-well plate containing 1.2 µL of lysis mix per well (0.095% Triton X-100, 1 U/µL Recombinant RNase Inhibitor, 2,5 mmol/L dNTPs, 2.5 µM Oligo-dT primer (5’-AAGCAGTGGTATCAACGCAGAGTACTTTTTTTTTTTTTTTTTTTTTTTTTTTT-TTVN-3’)), using FACSAria Fusion cytometer (BD Biosciences, Franklin Lakes, NJ, USA). scRNA-seq was performed using a modified SMART-seq2 method (V2.5) [[19](#_ENREF_19)]. Lysed cells were incubated for 3 minutes at 72 °C to complete lysis. The reverse transcription and template switching mix (10 U/µL Maxima H- reverse transcriptase [Thermo Fisher], 1.4 U/µL Recombinant RNase Inhibitor, 1X Maxima H- buffer [Thermo Fisher], 4,5 µmol/L template switching oligo [AAGCAGTGGTATCAACGCAGAGTACATrGrG+G], and 11,25% poly-ethylenglycol 8000 in nuclease-free water) was added and incubated for 90 minutes at 42 °C, followed by enzyme inactivation at 70 °C for 15 minutes. PCR amplification with 21 cycles was conducted by adding 3 µL KAPA HiFi HotStart ReadyMix (Roche, Basel, Switzerland) and 0.05 µL IS PCR primer (10 µmol/L) to each well and incubating according to the manufacturer’s protocol. After clean-up with 5 µL AmpureXP beads (Beckman-Coulter, Brea, CA, USA) at a 0.8 beads-to-sample ratio, quality control was conducted with Tapestation D5000 reagents (Agilent technologies, Santa Clara, CA, USA), and cDNA was diluted to 1–3 ng/µL. Fragmentation of full-length cDNA was performed using 1.2 µL Tn5 tagmentase in tagmentation buffer (10 mmol/L Tris-HCl pH 7.5, 10 mmol/L magnesium chloride, 50% dimethylformamide) with 0.4 µL cDNA, incubated for 10 minutes at 55°C. The reaction was stopped by adding 0.4 µL SDS. Barcoding oligos with Illumina sequencing barcodes were added, and library amplification was conducted by adding 2.7 µL KAPA HiFi HotStart ReadyMix (Roche) with 0.3 µL DMSO, followed by incubationaccording to the manufacturer’s protocol. After pooling and clean-up with AmpureXP beads (Beckman-Coulter) at a 0.9 beads-to-sample ratio, quality control was conducted with Tapestation D1000 reagents (Agilent). The final pooled library was diluted to 10 nmol/L for sequencing on the NextSeq 550 sequencing platform (High output, 75bp single-end, Illumina, San Diego, CA, USA).

### Data analysis of *in vivo* samples scRNA-seq samples

Quality control of the raw reads was conducted using FastQC, and adapter trimming was performed by the Next Generation Sequencing Unit of DKFZ. Alignment was carried out using the STARsolo method of the Spliced Transcripts Alignments to a Reference tool (STAR 2.7.11b, Alexander Dobin). Quality control, analysis, and plotting was performed using the R packages Seurat (version 5.1.0) [[20-24](#_ENREF_20)] and SingleCellExperiment (version 1.24.0) [[25](#_ENREF_25)]. Quality control followed the recommendations of the SeuratWrappers package (version 0.3.5) [[26](#_ENREF_26)] using the RunMiQC function with a posterior cutoff of 0.8, as well as the scuttle package (version 1.12.0) [[27](#_ENREF_27)] using the quickPerCellQC function. Genes expressed in fewer than two cells were discarded.Normalization, scaling, and centering were performed using the SCTransform function while accounting for potential confounders, including ribosomal and mitochondrial gene expression, cell cycle state, expression of housekeeping and dissociation-associated genes [[28-31](#_ENREF_28)] as well as the cell line and shRNA sequence. The scEWSR1::WT1 and scCACNA2D2 signatures were computed as follows: for scEWSR1::WT1 signature, the FindMarkers function was used to compare DOX-treated versus non-treated cells, identifying EWSR1::WT1-upregulated genes (log_2_FC > 0.25, *Padj* < 5×10^-5^, expression in > 10% of both DOX-treated and control cells) across both cell lines expressing shRNA against *EWSR1::WT1*; for scCACNA2D2 signature, the top 100 genes with the highest correlation coefficient with CACNA2D2 expression across all cell lines were selected. Signatures scores were calculated using the ssGSEA implementation of the irGSEA R package (version 3.2.5) [[32](#_ENREF_32)].

### Data analysis of scRNA-seq patient samples

The pre-processed and annotated scRNA-seq data from patient samples obtained from Henon et al. [[33](#_ENREF_33)] were analyzed using the ssGSEA implementation of the irGSEA R package and visualized using Seurat functions. All GEO accession codes for the scRNA-seq data analyzed in this study are provided in **Supplementary Table S9.**

### Gene expression microarray normalization

Publicly available and well-curated gene expression data of 654 samples, comprising 20 cancer entities and 929 normal tissue samples from 71 normal tissue types, were generated using the GeneChip™ Human Genome U133 Plus 2.0 Array microarrays (Applied Biosystems, Waltham, MA, USA) [[34](#_ENREF_34)]. These datasets were combined with 32 DSRCT samples [[35](#_ENREF_35)]. The GEO and ArrayExpress accession codes for each dataset analyzed are listed in **Supplementary Table S10**. All microarray .CEL files were simultaneously preprocessed (normalized) in R (version 4.3.0) using the affy package (version 1.78.2) [[36](#_ENREF_36)]. The Robust Multi-chip Average (RMA) algorithm, including background adjustment, quantile normalization, and summarization, was applied [[37](#_ENREF_37)]. For RMA normalization, custom brainarray Chip Description Files (CDF; ENTREZG, v25) were used, yielding one optimized probe set per gene [[38](#_ENREF_38)].

### Differential gene/protein expression analyses

Differential gene/protein expression analysis (DEG/DEP analysis) of microarray expression data or protein expression was performed using the limma package (version 3.56.2) [[39](#_ENREF_39)]. Preprocessed and normalized expression data were first log_2_ transformed. Then, DEG/DEP analysis was conducted by applying gene/protein-wise linear modeling, empirical bayes moderation, and statistical testing with limma. *P*-values were adjusted for multiple testing using false discovery rate (FDR) correction. DEG analysis of RNA-seq data was performed using the standard workflow of the DeSeq2 package (version 1.40.2) [[40](#_ENREF_40)]. Raw gene counts were retrieved from GEO (GEO accession code GSE212976), and lowly expressed genes were removed based on a count threshold < 10 across all samples analyzed. Then read counts were normalized using the DESeq2 method. Differential gene expression testing was conducted using the Wald test. *P*-values were adjusted for multiple testing using the Benjamini-Hochberg method.

### CACNA2D2 gene set and signature establishment

Correlation analysis using microarray gene expression data from 32 DSRCT patient samples was performed. The 2,873 coexpressed and 2,627 anti-coexpressed genes with *CACNA2D2* (|*r*| > 0.3; *P* < 0.05) were used to define the *CACNA2D2* gene set (**Supplementary Table S3**), and those genes with the highest *CACNA2D2* correlation scores (*n* = 100) defined the CACNA2D2 signature (**Supplementary Table S4**).

### Fast sample gene-set enrichment analyses (fGSEA) and single sample gene-set enrichment analyses (ssGSEA) and visualization

To identify enriched gene sets among genes coexpressed with *CACNA2D2*, genes were ranked according to their Pearson correlation coefficient, and a fast preranked gene-set enrichment analysis (fGSEA) was performed with 10,000 permutations in R using the fgsea package (version 1.26.0) [[41](#_ENREF_41)]. fGSEA was performed on the hallmark gene set (H) and curated gene sets, including canonical pathways from Reactome (C2:CP:REACTOME) from the Human Molecular Signatures Database (MSigDB, version v2023.2.Hs) [[42-45](#_ENREF_42)]. To visualize fGSEA results, enriched gene sets were filtered for significance (adjusted *P* < 0.01; |Normalized enrichment score| > 1.0). Heatmaps were annotated in R with ComplexHeatmap package (version 2.16.0) [[46](#_ENREF_46)].

### Western blotting

DSRCT cells containing DOX-inducible shRNAs were treated with DOX (1 µg/mL) for 96 hours. Whole cellular protein was extracted using RIPA buffer supplemented with 1X Halt™ Protease and Phosphatase Inhibitor Cocktail (Thermo Scientific). Proteins were separated on a 10% SDS-PAGE gel at 100 V and transferred onto PVDF membranes using the Trans-Blot Turbo Transfer System (BioRad). Membranes were incubated with mouse monoclonal anti-CACNA2D2 (1:1,000, sc-365911, Santa Cruz Biotechnology, Inc., Dallas, TX, USA), rabbit polyclonal anti-WT1 (1:500, sc-192, Santa Cruz), rabbit polyconal anti-EWSR1 (1:1000, #11910, Cell Signaling Technology Europe B.V. Leiden, The Netherlands) or rabbit monoclonal anti-GAPDH (1:1,000, #2118, Cell Signaling Technology). Then, membranes were incubated with horseradish peroxidase (HRP)-coupled anti-rabbit IgG (1:5,000, sc-2357, Santa Cruz) or anti-mouse IgG (1:5,000, A9044, Sigma-Aldrich). Proteins were detected using chemiluminescence and Immobilon Western HRP Substrat (Sigma-Aldrich).

### *In vivo* experiments

For subcutaneous experiments, 1×10^6^ DSRCT cells were resuspended in PBS and mixed with matrigel in 1:1 proportion (100 μL), and slowly injected subcutaneously into the right flank of 10 – 12 weeks old NOD/scid/gamma (NSG) mice as previously described [[47](#_ENREF_47)]. Tumor diameters were measured every second day with a caliper and tumor volume was calculated by the formula L × l^2^/2, where L is the length and l the width of the tumor. When the tumors reached an average volume of 80 mm^3^, mice were randomized into two groups. 96 hours prior to the pre-determined end of the experiment, mice were treated with either 2 mg/mL DOX dissolved in drinking water containing 5% sucrose to induce *in vivo* WT1 KD (DOX [+]), or 5% sucrose (control, DOX [-]). At the experimental endpoint or earlier if humane endpoints were reached (body weight loss of 20%, apathy, piloerection, self-isolation, aggression as a sign of pain, self-mutilation, motor abnormalities, as well as any other unphysiological or abnormal body posture, breathing difficulties, maximum tumor size of 15 mm in any direction, or an ulcerating tumor), mice were sacrificed by cervical dislocation. For orthotopic experiments, 1 × 10^6^ DSRCT cells were suspended in PBS, mixed 1:1 with matrigel (100 μL), and injected intraperitoneally into 10 – 12 weeks old NOD/scid/gamma (NSG) mice. Seven-weeks after injection, mice were randomized in two groups and treated with either 2 mg/mL DOX dissolved in drinking water containing 5% sucrose to induce *in vivo* WT1 KD (DOX [+]), or 5% sucrose (control, DOX [-]) for 96 hours. At the experimental endpoint or if the aforementioned humane endpoints were reached, mice were sacrificed by cervical dislocation. Then, tumors were extracted. A section of each tumor was snap frozen and reserved for RNA or protein extraction to confirm WT1 KD efficiency. Another section of each tumor was used for preparation of a single-cell suspension for scRNA-seq. The remaining tumor masses were fixed in 4% formalin and paraffin-embedded (FFPE) for (immuno)histological analysis. All experiments were approved by the government of North Baden and conducted in accordance with the ARRIVE guidelines, the European Community (86/609/EEC), and UKCCCR (guidelines for the welfare and use of animals in cancer research).

### Human samples and ethics approval

All samples analyzed in this study underwent rigorous morphological examination by reference pathologists and cancer-type specific molecular testing, including the identification of EWSR1::WT1 for DSRCTs whenever possible. DSRCT morphological mimics included alveolar soft part sarcoma (ASPS), Ewing sarcoma, ganglioneuroblastoma, leiomyosarcoma, liposarcoma, malignant fibrous histiocytoma, nephroblastoma, neuroblastoma, osteosarcoma, rhabdomyosarcoma, synovial sarcoma, gastrointestinal stromal tumor (GIST), mesothelioma, hepatoblastoma, and unspecified small round cell sarcomas. Open slides or tissue-microarrays (TMAs) from human FFPE or cryopreserved tissue samples were retrieved from the archives of the Institute of Pathology of the LMU Munich, the Charité Berlin, The Biobank of the Hospital Universitario Virgen del Rocío of Seville, the Hospital Gustave Roussy (Villejuif), the Bone Tumor Reference Center at the University of Basel, the University of Essen, the Cooperative Weichteilsarkom Studiengruppe (CWS) study center, the Klinikum Stuttgart (ethics committee from the Medical Faculty of the Eberhard-Karls University and University Hospital of Tübingen, approval no. 207/2022BO2), the Radboud University Medical Center, the Pathology Institute of the LMU Munich (approval no. 550-16 UE), and the University of Heidelberg (approval no. S-211/2021).

### Immunohistochemistry (IHC) and immunoreactivity scoring

Paraffin-embedded tissue sections (3–4 μm) were deparaffinized and rehydrated in distilled water. Antigen retrieval was performed using a steamer with Citrate Buffer (pH 6.0) for 20 minutes at 98 °C, followed by cooling to room temperature (RT). After rinsing with Tris Buffered Saline with 0.05% Tween 20 (TBS-T, Carl Roth GmbH + Co. KG, Karlsruhe, Germany), sections were blocked with BLOXALL blocking solution (Vector Laboratories, Newark, CA, USA) for 15 minutes at RT. Subsequently, they were incubated with 2.5% Horse Serum (Vector Laboratories) for 25 minutes at RT to minimize non-specific binding. Following two washes with TBS-T, sections were incubated with the primary monoclonal antibody against CACNA2D2 (1:3,000, sc-365911, Santa Cruz) in antibody diluent (Agilent technologies, Santa Clara, CA, USA) for 2 hours at 37°C. After three washes with TBS-T, sections were incubated with a secondary horseradish peroxidase (HRP)-coupled horse-anti-rabbit/mouse antibody (ImmPRESS HRP Universal PLUS Polymer Kit (Peroxidase, Horse Anti-Rabbit/Mouse IgG)) (Vector Laboratories) for 30 minutes at RT. After three washes with TBS-T, chromogen staining was performed using 3,3'-diaminobenzidine (DAB) for 10 minutes at RT. Sections were then rinsed in distilled water for 5 minutes followed by tap water for 10 minutes. Counterstaining was performed using Hemalum for 1 minute, followed by rinsing in tap water for 10 minutes. Finally, sections were dehydrated, cleared, and mounted with aqueous mounting media. Evaluation of CACNA2D2 immunoreactivity was conducted using a modified Immune Reactive Score (IRS) ranging from 0 – 12, adapted from the hormone receptor IRS scoring system, previously described and validated for Ewing sarcoma [[7](#_ENREF_7), [48-51](#_ENREF_48)]. The percentage of cells expressing the given antigen was scored and classified into five grades (grade 0 ≡ 0 – 19 %, 1 ≡ 20 – 39 %, 2 ≡ 40 – 59 %, 3 ≡ 60 – 79 % and 4 ≡ 80 – 100 %). In addition, the intensity of marker immunoreactivity was graded (grade 0 ≡ none, 1 ≡ low, 2 ≡ moderate and 3 ≡ strong). The final IRS was calculated as the product of these two grades.

### Automated sample preparation (autoSP3) for proteome profiling

Samples were prepared as previously described [[52](#_ENREF_52)], unless otherwise stated. In brief, cell pellets were resuspended in 75 µL SDS lysis buffer (4% SDS, 100 mmol/L Ammonium bicarbonate, pH 8.5). For cell lysis and protein extraction, samples were subjected to AFA-ultrasonication using the LE220R‐plus ultrasonicator (Covaris Ltd, Brighton, UK). Subsequently, protein concentrations were determined using the BCA Protein Assay Kit (Pierce, Thermo Fisher), and 20 µg of protein per sample was used as direct input for the autoSP3 protocol. The autoSP3 protocol, including protein clean-up, reduction and alkylation (using 10 mmol/L TCEP and 40 mol/L CAA at final concentration), and digestion (trypsin, enzyme:protein ratio of 1:20), was performed on the Bravo liquid handling system (Agilent Technologies) as previously described [[53](#_ENREF_53)] using the Paramagnetic beads for SP3 (Sera‐Mag Speed Beads A and B) (Fisher Scientific). The resulting peptides were directly frozen at − 80 °C until mass spectrometry (MS) acquisition.

### Mass spectrometry data acquisition and data processing

An equivalent amount of 200 ng peptides per sample was injected into the timsTOF Pro mass spectrometer (Bruker Daltonics, Billerica, MA, USA) coupled to an Easy nLC 1200 system (Thermo Scientific) fitted with an analytical column (Aurora Column with CSI fitting, C18, 1.6 μm, 75 μm x 25 cm) (Ionopticks, Collingwood, Australia). The elution gradient was set to 80 minutes at a flow rate of 300 nl/minute using solvent A (0.1% formic acid in ULCM grade water) and solvent B (0.1% formic acid in 80% acetonitrile and 19.9% ULCM grade water). Data were acquired in DIA-PASEF mode. The full scan MS spectra were set to a mass range 100 to 1,700 m/z and 1/k0 range from 0.65 to 1.42 V*s/cm^2^ with a 100 ms ramp time. The duty cycle was locked at 100%, the ion polarity was set to positive, and TIMS mode was enabled. Collision energy was set to 1/k0 range from 0.65 to 1.42 V*s/cm^2^. For the DIA scans, a custom isolation window pattern was optimized, covering the precursor range of 377 to 1,194 m/z, mobility range 1/k0 range from 0.67 to 1.39 V*s/cm^2^, and cycle time estimate of 1.58 s. The resulting raw files were processed with DIA-NN software (version 1.8.1) [[54](#_ENREF_54)] using default settings unless otherwise stated. Trypsin/P was selected as digesting enzyme, with a maximum of two missed cleavages. N-term M excision, C carbamidomethylation, and oxidation were set as fixed and variable modifications. The match-between-runs (MBR) function was allowed. The DIA-NN search was performed using the *H.sapiens* Uniprot database (reviewed only, downloaded on 30.03.2021), and an in silico spectral library was generated by FragPipe using the previously mentioned fasta file.

### UMAP clustering of methylation array data

Preprocessed beta values from methylation arrays of reference cases from the Sarcoma classifier [[55](#_ENREF_55)] were imported into R. Dimensionality reduction was performed using Uniform Manifold Approximation and Projection (UMAP) with the umap R package (version 0.2.10.0) [[56](#_ENREF_56), [57](#_ENREF_57)]. UMAP parameters were set to 30 neighbors and a minimum distance of 0.1. Clustering results were visualized using color-coded labels for different entities in a UMAP plot generated with the ggplot2 R package (version 3.4.4) [[58](#_ENREF_58)]. Genes selected for analysis included *CACNA2D2*, other described EWSR1::WT1-regulated genes [[4](#_ENREF_4), [8](#_ENREF_8), [59-64](#_ENREF_59)] and *IQCG.*

### Statistical analyses

Unless otherwise specified, statistical data analysis was performed using PRISM 9 (GraphPad Software Inc., CA, USA) on the raw data. Unless otherwise specified in the figure legends or the respective methods section, comparisons between two groups in functional *in vitro* experiments were conducted using an unpaired two-sided Mann-Whitney test. Pearson correlation analysis was performed in R using the Stats package (version 4.3.0) and visualized in correlation matrices plotted with the corrplot package (version 0.92). Unless otherwise specified in the figure legends, data are presented as box-dot plots, with horizontal bars representing means and whiskers indicating the standard error of the mean (SEM). Sample size for all *in vitro* experiments was chosen empirically. For *in vivo* experiments, sample size was predetermined using power calculations with *β* = 0.8 and *α* < 0.05 based on preliminary data and in compliance with the 3R principles (replacement, reduction, refinement). *P*-values < 0.05 were considered as statistically significant. Unless otherwise specified in the figure legends, all *P*-values were estimated using nonparametric two-sided statistical tests.

## Supplementary references

1. Markides CSA, Coil DR, Luong LH, Mendoza J, Kozielski T, Vardeman D, et al. Desmoplastic small round cell tumor (DSRCT) xenografts and tissue culture lines: Establishment and initial characterization. Oncology Letters. 2013;5(5):1453-6.

2. Wiederschain D, Wee S, Chen L, Loo A, Yang G, Huang A, et al. Single-vector inducible lentiviral RNAi system for oncology target validation. Cell Cycle (Georgetown, Tex). 2009;8(3):498-504.

3. Musa J, Cidre-Aranaz F, Aynaud M-M, Orth MF, Knott MML, Mirabeau O, et al. Cooperation of cancer drivers with regulatory germline variants shapes clinical outcomes. Nature Communications. 2019;10(1):4128.

4. Gedminas JM, Chasse MH, Mcbrairty M, Beddows I, Kitchen-Goosen SM, Grohar PJ. Desmoplastic small round cell tumor is dependent on the EWS-WT1 transcription factor. Oncogenesis. 2020;9(4).

5. Los GV, Encell LP, McDougall MG, Hartzell DD, Karassina N, Zimprich C, et al. HaloTag: A Novel Protein Labeling Technology for Cell Imaging and Protein Analysis. ACS Chemical Biology. 2008;3(6):373-82.

6. Schwinn MK, Machleidt T, Zimmerman K, Eggers CT, Dixon AS, Hurst R, et al. CRISPR-Mediated Tagging of Endogenous Proteins with a Luminescent Peptide. ACS Chemical Biology. 2018;13(2):467-74.

7. Cidre-Aranaz F, Li J, Hölting TLB, Orth MF, Imle R, Kutschmann S, et al. Integrative gene network and functional analyses identify a prognostically relevant key regulator of metastasis in Ewing sarcoma. Molecular Cancer. 2022;21(1):1.

8. Hingorani P, Dinu V, Zhang X, Lei H, Shern JF, Park J, et al. Transcriptome analysis of desmoplastic small round cell tumors identifies actionable therapeutic targets: a report from the Children’s Oncology Group. Scientific Reports. 2020;10(1).

9. Heinz S, Benner C, Spann N, Bertolino E, Lin YC, Laslo P, et al. Simple combinations of lineage-determining transcription factors prime cis-regulatory elements required for macrophage and B cell identities. Molecular Cell. 2010;38(4):576-89.

10. Church DM, Schneider VA, Graves T, Auger K, Cunningham F, Bouk N, et al. Modernizing Reference Genome Assemblies. PLOS Biology. 2011;9(7):e1001091.

11. Grant CE, Bailey TL, Noble WS. FIMO: scanning for occurrences of a given motif. Bioinformatics (Oxford, England). 2011;27(7):1017-8.

12. Sayers EW, Bolton EE, Brister JR, Canese K, Chan J, Comeau DC, et al. Database resources of the national center for biotechnology information. Nucleic Acids Research. 2022;50(D1):D20-D6.

13. Li H. Aligning sequence reads, clone sequences and assembly contigs with BWA-MEM. 2013.

14. Danecek P, Bonfield JK, Liddle J, Marshall J, Ohan V, Pollard MO, et al. Twelve years of SAMtools and BCFtools. GigaScience. 2021;10(2):giab008.

15. Zhang Y, Liu T, Meyer CA, Eeckhoute J, Johnson DS, Bernstein BE, et al. Model-based Analysis of ChIP-Seq (MACS). Genome Biology. 2008;9(9):R137.

16. Whyte WA, Orlando DA, Hnisz D, Abraham BJ, Lin CY, Kagey MH, et al. Master Transcription Factors and Mediator Establish Super-Enhancers at Key Cell Identity Genes. Cell. 2013;153(2):307-19.

17. Lovén J, Hoke HA, Lin CY, Lau A, Orlando DA, Vakoc CR, et al. Selective Inhibition of Tumor Oncogenes by Disruption of Super-Enhancers. Cell. 2013;153(2):320-34.

18. Truong DD, Lamhamedi-Cherradi S-E, Porter RW, Krishnan S, Swaminathan J, Gibson A, et al. Dissociation protocols used for sarcoma tissues bias the transcriptome observed in single-cell and single-nucleus RNA sequencing. BMC Cancer. 2023;23(1):488.

19. Picelli S, Faridani OR, Björklund AK, Winberg G, Sagasser S, Sandberg R. Full-length RNA-seq from single cells using Smart-seq2. Nature Protocols. 2014;9(1):171-81.

20. Hao Y, Stuart T, Kowalski MH, Choudhary S, Hoffman P, Hartman A, et al. Dictionary learning for integrative, multimodal and scalable single-cell analysis. Nature Biotechnology. 2024;42(2):293-304.

21. Hao Y, Hao S, Andersen-Nissen E, Mauck WM, Zheng S, Butler A, et al. Integrated analysis of multimodal single-cell data. Cell. 2021;184(13):3573-87.e29.

22. Stuart T, Butler A, Hoffman P, Hafemeister C, Papalexi E, Mauck WM, et al. Comprehensive Integration of Single-Cell Data. Cell. 2019;177(7):1888-902.e21.

23. Butler A, Hoffman P, Smibert P, Papalexi E, Satija R. Integrating single-cell transcriptomic data across different conditions, technologies, and species. Nature Biotechnology. 2018;36(5):411-20.

24. Satija R, Farrell JA, Gennert D, Schier AF, Regev A. Spatial reconstruction of single-cell gene expression data. Nature Biotechnology. 2015;33(5):495-502.

25. Amezquita RA, Lun ATL, Becht E, Carey VJ, Carpp LN, Geistlinger L, et al. Orchestrating single-cell analysis with Bioconductor. Nature Methods. 2020;17(2):137-45.

26. Hippen AA, Falco MM, Weber LM, Erkan EP, Zhang K, Doherty JA, et al. miQC: An adaptive probabilistic framework for quality control of single-cell RNA-sequencing data. 2021.

27. McCarthy DJ, Campbell KR, Lun ATL, Wills QF. Scater: pre-processing, quality control, normalization and visualization of single-cell RNA-seq data in R. Bioinformatics. 2017;33(8):1179-86.

28. Eisenberg E, Levanon EY. Human housekeeping genes, revisited. Trends in genetics: TIG. 2013;29(10):569-74.

29. Adam M, Potter AS, Potter SS. Psychrophilic proteases dramatically reduce single-cell RNA-seq artifacts: a molecular atlas of kidney development. Development (Cambridge, England). 2017;144(19):3625-32.

30. van den Brink SC, Sage F, Vértesy Á, Spanjaard B, Peterson-Maduro J, Baron CS, et al. Single-cell sequencing reveals dissociation-induced gene expression in tissue subpopulations. Nature Methods. 2017;14(10):935-6.

31. Denisenko E, Guo BB, Jones M, Hou R, de Kock L, Lassmann T, et al. Systematic assessment of tissue dissociation and storage biases in single-cell and single-nucleus RNA-seq workflows. Genome Biology. 2020;21(1):130.

32. Fan C, Chen F, Chen Y, Huang L, Wang M, Liu Y, et al. irGSEA: the integration of single-cell rank-based gene set enrichment analysis. Briefings in Bioinformatics. 2024;25(4):bbae243.

33. Henon C, Vibert J, Eychenne T, Gruel N, Colmet-Daage L, Ngo C, et al. Single-cell multiomics profiling reveals heterogeneous transcriptional programs and microenvironment in DSRCTs. Cell Reports Medicine. 2024;5(6):101582.

34. Baldauf MC, Orth MF, Dallmayer M, Marchetto A, Gerke JS, Rubio RA, et al. Robust diagnosis of Ewing sarcoma by immunohistochemical detection of super-enhancer-driven EWSR1-ETS targets. Oncotarget. 2017;9(2):1587-601.

35. Surdez D, Benetkiewicz M, Perrin V, Han Z-Y, Pierron G, Ballet S, et al. Targeting the EWSR1-FLI1 Oncogene-Induced Protein Kinase PKC-β Abolishes Ewing Sarcoma Growth. Cancer Research. 2012;72(17):4494-503.

36. Gautier L, Cope L, Bolstad BM, Irizarry RA. affy--analysis of Affymetrix GeneChip data at the probe level. Bioinformatics (Oxford, England). 2004;20(3):307-15.

37. Irizarry RA, Hobbs B, Collin F, Beazer-Barclay YD, Antonellis KJ, Scherf U, et al. Exploration, normalization, and summaries of high density oligonucleotide array probe level data. Biostatistics (Oxford, England). 2003;4(2):249-64.

38. Dai M, Wang P, Boyd AD, Kostov G, Athey B, Jones EG, et al. Evolving gene/transcript definitions significantly alter the interpretation of GeneChip data. Nucleic Acids Research. 2005;33(20):e175.

39. Ritchie ME, Phipson B, Wu D, Hu Y, Law CW, Shi W, et al. limma powers differential expression analyses for RNA-sequencing and microarray studies. Nucleic Acids Research. 2015;43(7):e47.

40. Love MI, Huber W, Anders S. Moderated estimation of fold change and dispersion for RNA-seq data with DESeq2. Genome Biology. 2014;15(12):550.

41. Korotkevich G, Sukhov V, Budin N, Shpak B, Artyomov MN, Sergushichev A. Fast gene set enrichment analysis. bioRxiv. 2021:060012.

42. Subramanian A, Tamayo P, Mootha VK, Mukherjee S, Ebert BL, Gillette MA, et al. Gene set enrichment analysis: A knowledge-based approach for interpreting genome-wide expression profiles. Proceedings of the National Academy of Sciences. 2005;102(43):15545-50.

43. Liberzon A, Birger C, Thorvaldsdóttir H, Ghandi M, Mesirov JP, Tamayo P. The Molecular Signatures Database (MSigDB) hallmark gene set collection. Cell Systems. 2015;1(6):417-25.

44. Milacic M, Beavers D, Conley P, Gong C, Gillespie M, Griss J, et al. The Reactome Pathway Knowledgebase 2024. Nucleic Acids Research. 2024;52(D1):D672-D8.

45. Liberzon A, Subramanian A, Pinchback R, Thorvaldsdóttir H, Tamayo P, Mesirov JP. Molecular signatures database (MSigDB) 3.0. Bioinformatics. 2011;27(12):1739-40.

46. Gu Z, Eils R, Schlesner M. Complex heatmaps reveal patterns and correlations in multidimensional genomic data. Bioinformatics. 2016;32(18):2847-9.

47. Ewing Sarcoma: Methods and Protocols. New York, NY: Springer US; 2021 2021.

48. Baldauf MC, Gerke JS, Kirschner A, Blaeschke F, Effenberger M, Schober K, et al. Systematic identification of cancer-specific MHC-binding peptides with RAVEN. Oncoimmunology. 2018;7(9):e1481558.

49. Marchetto A, Ohmura S, Orth MF, Knott MML, Colombo MV, Arrigoni C, et al. Oncogenic hijacking of a developmental transcription factor evokes vulnerability toward oxidative stress in Ewing sarcoma. Nature Communications. 2020;11(1):2423.

50. Dallmayer M, Li J, Ohmura S, Alba Rubio R, Baldauf MC, Hölting TLB, et al. Targeting the CALCB/RAMP1 axis inhibits growth of Ewing sarcoma. Cell Death & Disease. 2019;10(2):116.

51. Ohmura S, Marchetto A, Orth MF, Li J, Jabar S, Ranft A, et al. Translational evidence for RRM2 as a prognostic biomarker and therapeutic target in Ewing sarcoma. Molecular Cancer. 2021;20(1):97.

52. Müller T, Kalxdorf M, Longuespée R, Kazdal DN, Stenzinger A, Krijgsveld J. Automated sample preparation with SP3 for low‐input clinical proteomics. Molecular Systems Biology. 2020;16(1):e9111.

53. Müller T, Cremonini MA, Kliewer G, Krijgsveld J. Automated Sample Preparation for Mass Spectrometry-Based Clinical Proteomics. In: Gevaert K, editor. Mass Spectrometry-Based Proteomics. New York, NY: Springer US; 2023. p. 181-211.

54. Demichev V, Messner CB, Vernardis SI, Lilley KS, Ralser M. DIA-NN: neural networks and interference correction enable deep proteome coverage in high throughput. Nature Methods. 2020;17(1):41-4.

55. Koelsche C, Schrimpf D, Stichel D, Sill M, Sahm F, Reuss DE, et al. Sarcoma classification by DNA methylation profiling. Nature Communications. 2021;12(1):498.

56. McInnes L, Healy J, Melville J. UMAP: Uniform Manifold Approximation and Projection for Dimension Reduction. 2020.

57. McInnes L, Healy J, Saul N, Großberger L. UMAP: Uniform Manifold Approximation and Projection. Journal of Open Source Software. 2018;3(29):861.

58. Wickham H. Data Analysis. ggplot2. Cham: Springer International Publishing; 2016. p. 189-201.

59. Magrath JW, Sampath SS, Flinchum DA, Hartono AB, Goldberg IN, Boehling JR, et al. Comprehensive Transcriptomic Analysis of EWSR1::WT1 Targets Identifies CDK4/6 Inhibitors as an Effective Therapy for Desmoplastic Small Round Cell Tumors. Cancer Research. 2024;84(9):1426-42.

60. Ogura K, Somwar R, Hmeljak J, Magnan H, Benayed R, Momeni Boroujeni A, et al. Therapeutic Potential of NTRK3 Inhibition in Desmoplastic Small Round Cell Tumor. Clinical Cancer Research. 2020:clincanres.2585.

61. Palmer RE, Lee SB, Wong JC, Reynolds PA, Zhang H, Truong V, et al. Induction of BAIAP3 by the EWS-WT1 chimeric fusion implicates regulated exocytosis in tumorigenesis. Cancer Cell. 2002;2(6):497-505.

62. Li H, Smolen GA, Beers LF, Xia L, Gerald W, Wang J, et al. Adenosine Transporter ENT4 Is a Direct Target of EWS/WT1 Translocation Product and Is Highly Expressed in Desmoplastic Small Round Cell Tumor. PLoS ONE. 2008;3(6):e2353.

63. Saito T. EWS-WT1 Chimeric Protein in Desmoplastic Small Round Cell Tumor is a Potent Transactivator of FGFR4. Journal of Cancer Science & Therapy. 2012;04(10).

64. Negri T, Brich S, Bozzi F, Volpi CV, Gualeni AV, Stacchiotti S, et al. New transcriptional-based insights into the pathogenesis of desmoplastic small round cell tumors (DSRCTs). Oncotarget. 2017;8(20):32492-504.

## Supplementary Figure Legends


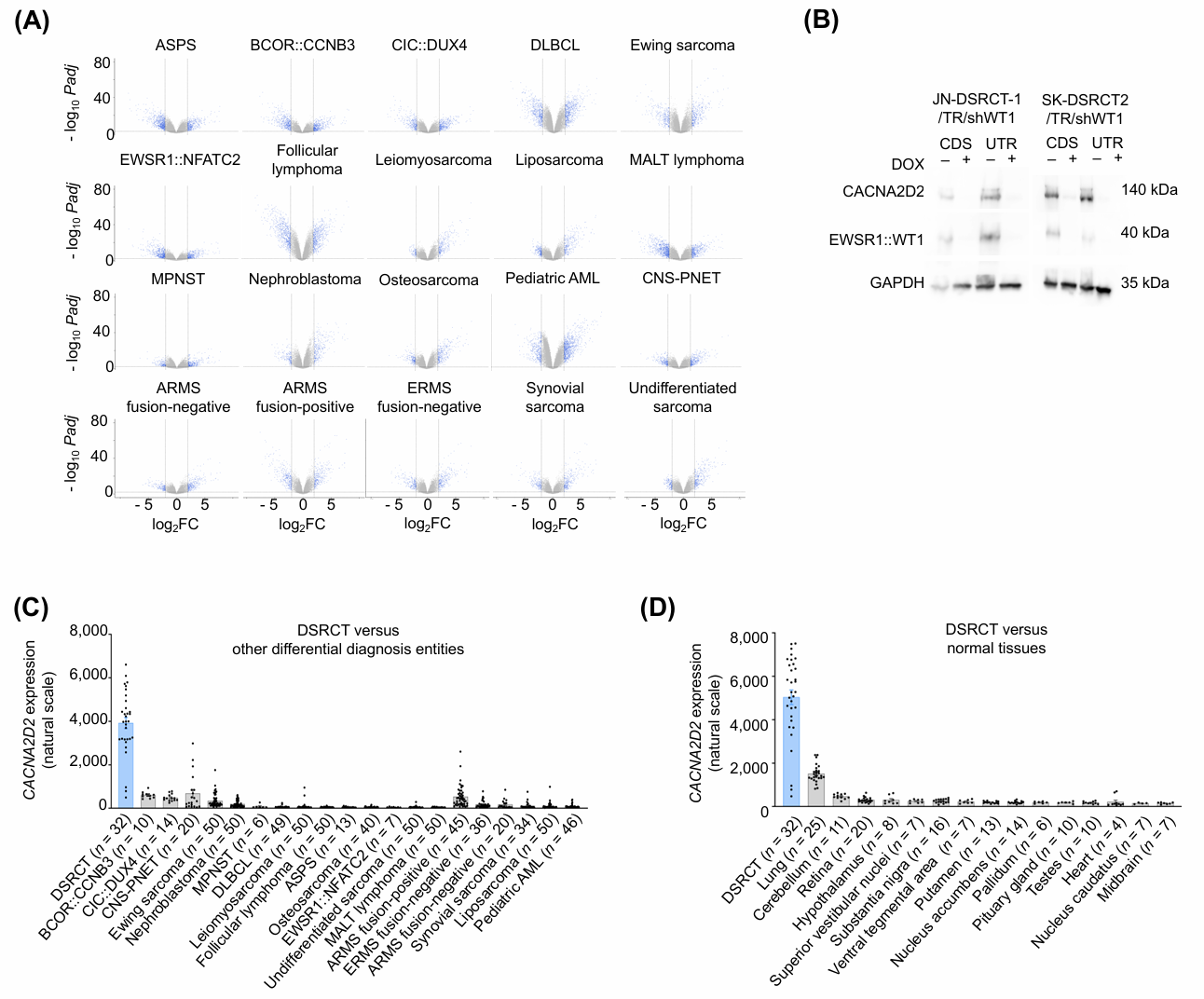


### Supplementary Figure S1. CACNA2D2 is significantly overexpressed in DSRCT compared to cancer entities of differential diagnostic relevance.

**(A)** Volcano plots depicting results of pairwise differential gene expression analysis of microarray mRNA expression profiles from primary DSRCT tumor samples and the indicated tumor entity. Blue dots represent genes with |log_2_FC| > 2.5 and *Padj* < 0.01 (Benjamini-Hochberg corrected).

**(B)** Western blot using antibodies against CACNA2D2, WT1, and GAPDH (loading control) in DSRCT tumor samples derived from orthotopic xenografts of JN-DSRCT-1 and SK-DSRCT2 cell lines expressing a DOX-inducible shRNA-mediated KD system for EWSR1::WT1. Mice were treated either with DOX or sucrose (control) for 96 hours.

**(C)** Bar plot showing *CACNA2D2* mRNA expression in 32 DSRCT patient samples compared to 654 patient samples from 20 different cancer entities.

**(D)** Bar plot showing *CACNA2D2* mRNA expression in 165 samples from the 15 normal tissue types with the highest *CACNA2D2* expression, compared to 32 DSRCT patient samples. Horizontal bars in bar plots represent mean expression levels, with whiskers indicating SEM. The number of analysed samples is given in parentheses. Unpaired two-sided Mann-Whitney test.

Abbreviations: AML, acute myeloid leukaemia; Padj, adjusted P-value; (Padj), ARMS, alveolar rhabdomyosarcoma; ASPS, alveolar soft part sarcoma; BCOR::CCNB3, BCOR::CCNB3 rearranged sarcomas; CACNA2D2, Calcium voltage-gated channel auxiliary subunit alpha2delta 2 gene; CIC::DUX4, CIC::DUX4 rearranged sarcomas; CDS, coding sequence; DLBCL, diffuse large B-cell lymphoma; DOX, doxycycline; ERMS, embryonal rhabdomyosarcoma; EWSR1::NFATC2, EWSR1::NFATC2 rearranged sarcomas; EWSR1::WT1, fusion protein of EWSR1 and WT1 proteins; GAPDH, Glyceraldehyde-3-phosphate dehydrogenase; JN-DSRCT-1/TR/shWT1, JN-DSRCT-1 cell line stably expressing a doxycycline-inducible shRNA expression cassette targeting EWSR1::WT1 mRNA; kDa, kilodalton; KD, knock down; log2FC, log2 fold change; MPNST, malignant peripheral nerve sheath tumor; MALT, mucosa-associated lymphoid tissue lymphoma; CNS-PNET, primitive neuroectodermal tumor arising in the central nervous system; shRNA, short hairpin RNA; shWT1, shRNA targeting EWSR1::WT1 mRNA; SK-DSRCT2/TR/shWT1, SK-DSRCT2 cell line stably expressing a doxycycline-inducible shRNA expression cassette targeting EWSR1::WT1 mRNA; SEM, standard error of the mean; UTR, untranslated region.


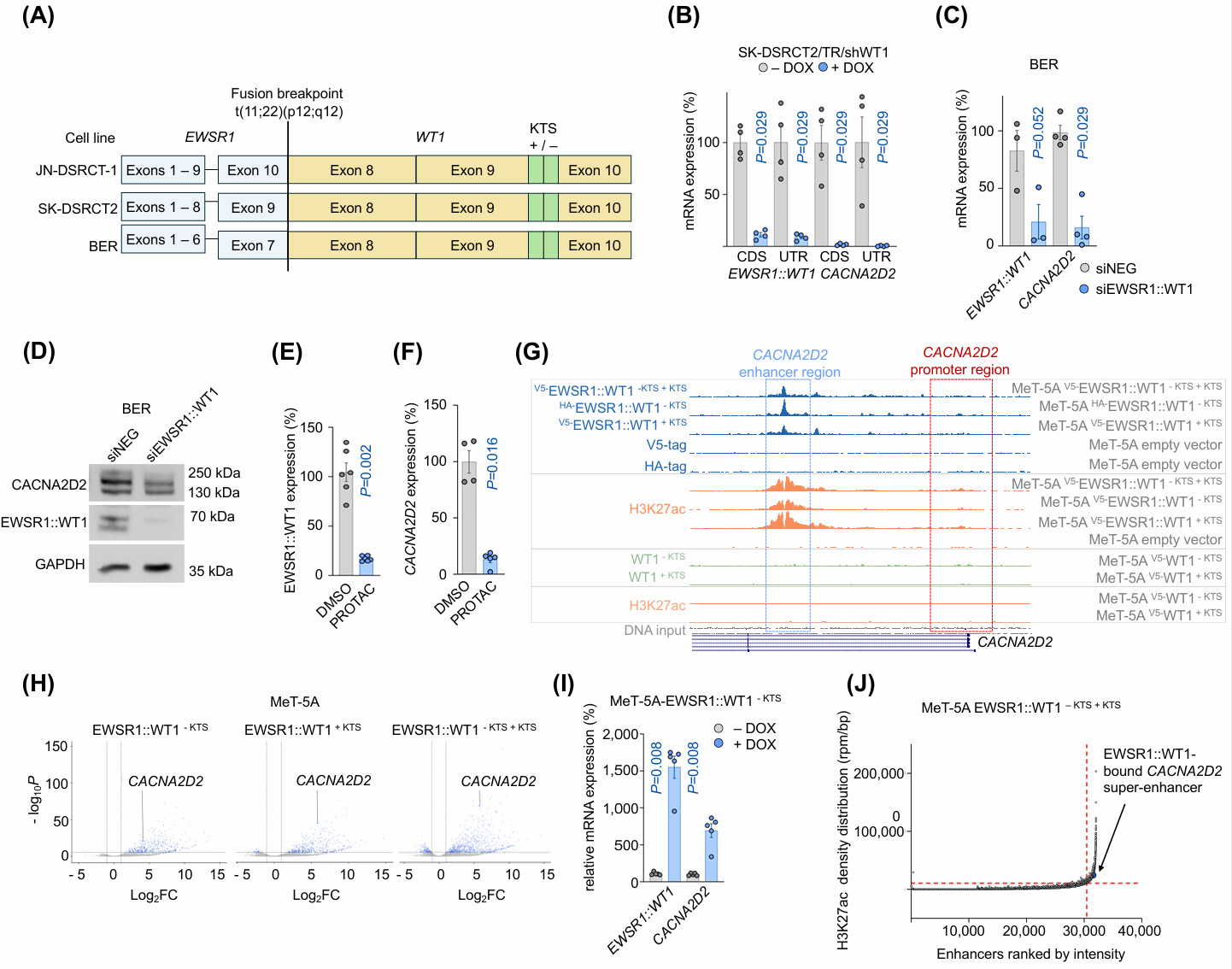


### Supplementary Figure S2. *CACNA2D2* is directly regulated by an EWSR1::WT1-bound super-enhancer.

**(A)** Diagram depicting the different EWSR1::WT1 transcript variants expressed in JN-DSRCT-1, SK-DSRCT2, and BER DSRCT cell lines. The fusion breakpoint spans different exons of the *EWSR1* gene. The *WT1* gene either lacks (- KTS) or contains (+ KTS) the KTS motif at the junction between exons 9 and 10.

**(B)** Bar plot showing relative mRNA expression levels of *EWSR1::WT1* and *CACNA2D2,* quantified by qRT-PCR, in SK-DSRCT2 cell line expressing a DOX-inducible shRNA-mediated KD of EWSR1::WT1 for 96 hours. *n* = 4 biologically independent experiments**.**

**(C)** Bar plot showing relative mRNA expression levels of *EWSR1::WT1* and *CACNA2D2*, quantified by qRT-PCR, in the BER DSRCT cell line transfected with siRNAs targeting *EWSR1::WT1* (siEWSR1::WT1) or a negative control (siNEG) for 48 hours. *n* ≥ 3 biologically independent experiments.

**(D)** Western blot using antibodies against CACNA2D2, EWSR1 (-WT1), and GAPDH (loading control) in BER cells transfected as in **Figure 1F**.

**(E)** Bar plot showing relative expression levels of the *EWSR1::WT1-HaloTag-HiBiT* transcript in SK-DSRCT2-endo-WT1-HaloTag, as described in **Figure 1F**. *n* = 6 biologically independent experiments.

**(F)** Bar plot showing relative expression levels of *CACNA2D2* transcript in SK-DSRCT2-endo-WT1-HaloTag cells, as described in **Figure 1F**. *n* ≥ 4 biologically independent experiments.

**(G)** UCSC genome browser showing the epigenetic profile of the *CACNA2D2* locus (chr3:50,506,517 – 50,548,707) in MeT-5A human mesothelial cells expressing different isoforms of V5- or HA-tagged wild-type WT1, EWSR1::WT1, or an empty control vector. ChIP-seq data derived from GSE212977 are depicted for EWSR1::WT1 (blue), H3K27ac (orange), WT1 (green), DNA input (gray). The *CACNA2D2* promoter and enhancer regions are highlighted with dotted rectangles.

**(H)** Volcano plots depicting results of differential gene expression analysis of RNA-seq data from MeT-5A mesothelial cells expressing different EWSR1::WT1 isoforms (GSE212979). Blue dots represent genes with |log_2_FC| > 1.0 and *Padj* < 0.01 (Benjamini-Hochberg corrected).

**(I)** Bar plot showing relative mRNA expression levels of ectopically overexpressed *EWSR1::WT1* and endogenous *CACNA2D2*, quantified by qRT-PCR, in MeT-5A mesothelial cells carrying a DOX-inducible EWSR1::WT1 expression cassette. Cells were treated with DOX for 96 hours. *n* = 5 biologically independent experiments.

**(J)** x-y scatterplot showing H3K27ac signal density (reads per million mapped reads per base) at active enhancer sites in MeT-5A cells (GSE212977) expressing EWSR1::WT1 ^– KTS + KTS^, ranked by normalized intensity from low to high. The horizontal dashed red line indicates a cut-off value of 10,645.1 for the identification of super-enhancers (*n* = 1,594, as indicated by the vertical dashed red line). Horizontal bars in bar plots represent mean expression levels, and whiskers indicate SEM. The number of analysed samples is given in parentheses. Unpaired two-sided Mann-Whitney test.

Abbreviations: Padj, Adjusted P-value; KTS, amino acid motif containing lysine-threonine-serine amino acids within WT1 CDS; CACNA2D2, Calcium voltage-gated channel auxiliary subunit alpha2delta 2 gene; ChIP-seq, chromatin immunoprecipitation followed by sequencing; chr3, chromosome 3; CDS, coding sequence; DMSO, dimethyl sulfoxide; DOX, doxycycline; EWSR1, EWS RNA binding protein 1; EWSR1::WT1-HaloTag-HiBiT, EWSR1::WT1 protein fused to HaloTag-HiBiT protein; EWSR1::WT1, fusion protein of EWSR1 and WT1 proteins; GAPDH, Glyceraldehyde-3-phosphate dehydrogenase; HA, HA-tag; H3K27ac, histone H3 lysine 27 acetylation; kDa, kilodalton; KD, knock down; log2FC, log2 fold change; siNEG, non-targeting control siRNA; PROTAC, proteolysis targeting chimera; RNA-seq, RNA-sequencing; qRT-PCR, quantitative real-time polymerase chain reaction; rpm/bp, reads per million per base pair; shRNA, short-hairpin RNA; siEWSR1::WT1, siRNAs targeting EWSR1:WT1 mRNA; SK-DSRCT2-endo-WT1-HaloTag, SK-DSRCT2 cell line expressing endogenous CACNA2D2 protein fused to HaloTag protein; SK-DSRCT2/TR/shWT1, SK-DSRCT2 cell line stably expressing a doxycycline-inducible shRNA expression cassette targeting EWSR1::WT1 mRNA; SEM, standard error of the mean; UTR, untranslated region; V5, V5-tag; WT1, Wilms tumor protein.


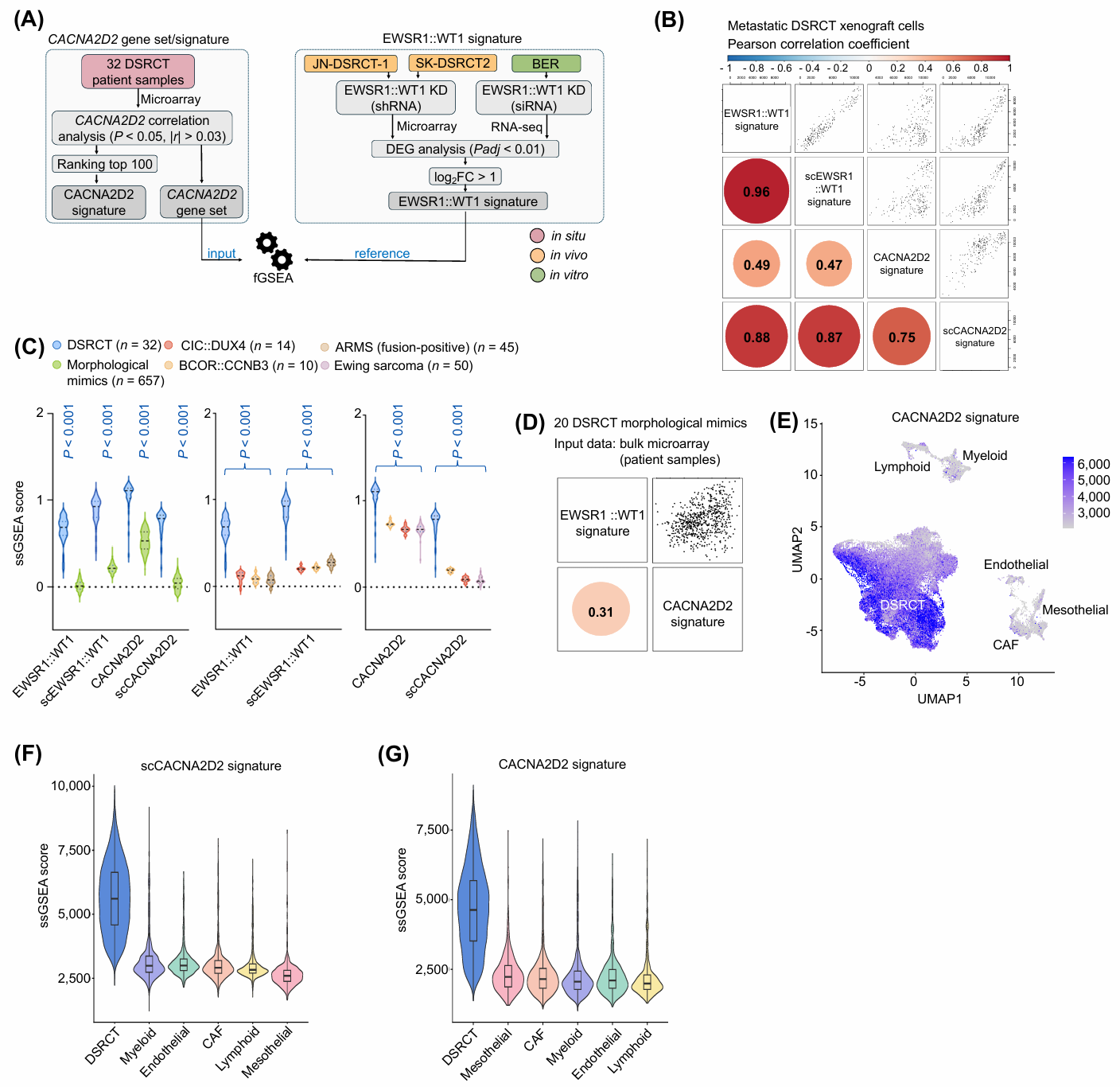


### Supplementary Figure S3. CACNA2D2 is a key component of the EWSR1::WT1 oncogenic signature.

**(A)** Diagram illustrating the workflow for establishing the CACNA2D2 signature, the *CACNA2D2* gene set, and the EWSR1::WT1 signature for fGSEA using Pearson correlation and DEG analysis, respectively. Colors indicate the origin of the samples (*in situ, in vivo, in vitro*).

**(B)** x-y scatter plots illustrating the correlation of ssGSEA enrichment scores between (sc)EWSR1::WT1 and (sc)CACNA2D2 signatures in single metastatic DSRCT cells from orthotopic xenografts. The numbers and colors in the correlation matrix represent Pearson correlation values for each signature enrichment.

**(C)** Left: violin plot comparing ssGSEA scores for (sc)EWSR1::WT1 and (sc)CACNA2D2 signatures in 32 DSRCT patient samples versus all other DSRCT-morphological mimics analyzed in **Figure 1H**. Middle: violin plot comparing ssGSEA enrichment scores for (sc)EWSR1::WT1 in 32 DSRCT patient samples versus the top three scoring entities: BCOR::CCNB3, CIC::DUX4, and fusion-positive ARMS. Right: violin plot comparing ssGSEA enrichment scores for the (sc)CACNA2D2 signature in 32 DSRCT patient samples versus the top three scoring entities: BCOR::CCNB3, CIC::DUX4, and Ewing sarcoma. Dotted black lines represent the median, and dotted blue lines indicate quartiles. Unpaired two-sided Mann-Whitney test.

**(D)** Correlation matrix showing ssGSEA enrichment scores of EWSR1::WT1 and CACNA2D2 signatures across all 20 cancer entities of DSRCT differential diagnosis.

**(E)** UMAP plot of single-cell RNA-seq analysis of merged and integrated data from eleven samples, comprising tumor-derived and normal cells from four DSRCT patients (GSE263523), using the CACNA2D2 signature. The color gradient represents the ssGSEA score for CACNA2D2 signature enrichment.

**(F)** Violin plot depicting scCACNA2D2 signature scores for DSRCT and other normal cell types present in the patient samples. The horizontal bar represents the median.

**(G)** Violin plot depicting CACNA2D2 signature scores for DSRCT and other normal cell types present in the patient samples. The horizontal bar represents the median.

Abbreviations: ARMS, Adjusted P-value; ARMS, alveolar rhabdomyosarcoma; BCOR, BCL-6 Corepressor; BCOR::CCNB3, BCOR::CCNB3 rearranged sarcomas; CACNA2D2, Calcium voltage-gated channel auxiliary subunit alpha2delta 2 gene; CAFs, cancer-associated fibroblasts; CIC , Capicua transcriptional repressor; CIC::DUX4, CIC::DUX4 rearranged sarcomas; CCNB3, Cyclin B3; DEG, differential gene expression; DOX, doxycycline; DUX4, Double homeobox 4; fGSEA, fast gene set enrichment analysis; EWSR1::WT1, fusion protein of EWSR1 and WT1 proteins; KD, knock down; log2FC, log2 fold change; RNA-seq, RNA-sequencing; shRNA, short-hairpin RNA; scCACNA2D2, single-cell data derived CACNA2D2 signature; scEWSR1::WT1, single-cell data derived EWSR1::WT1 signature; ssGSEA, single-sample gene set enrichment analysis; siRNA, small-interfering RNA; UMAP, uniform manifold approximation and projection.


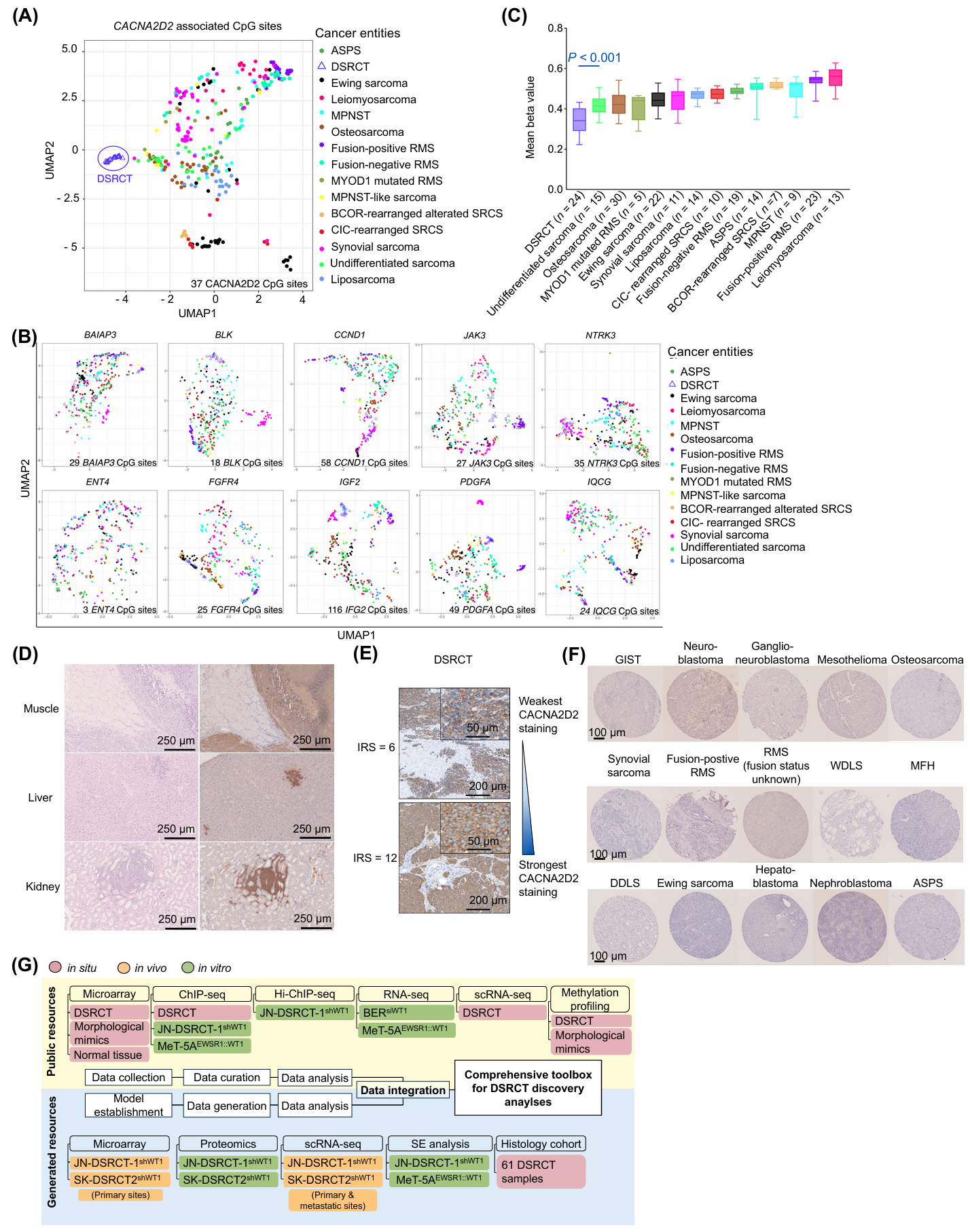


### Supplementary Figure S4. High CACNA2D2 expression enables robust DSRCT diagnosis by IHC.

**(A)** UMAP plot showing 14 sarcoma entities, including DSRCT, clustered based on their *CACNA2D2* methylation status.

**(B)** UMAP plots showing 14 sarcoma entities, including DSRCT, clustered according to the CpG sites associated with each indicated gene. The number of analyzed CpG sites is shown in the bottom right corner of each plot.

**(C)** Box plot showing the mean methylation of *CACNA2D2*-associated CpG sites across 14 sarcoma entities, including DSRCT. Boxes represent the minimum and maximum values, while the horizontal line indicates the median. The number of analyzed samples is given in parentheses. Unpaired two-sided Mann-Whitney test**.**

**(D)** Representative histological images of CACNA2D2 IHC in micrometastases within internal organs (muscle, liver, and kidney) from murine DSRCT orthotopic xenografts. A brown chromogen was used.

**(E)** Representative histological images of CACNA2D2 IHC in DSRCT patient samples with IRS values of 6 or 12, illustrating the range of staining intensities observed in DSRCT. A brown chromogen was used.

**(F)** Representative histological images of CACNA2D2 IHC in TMA cores from different cancer entities. A brown chromogen was used.

**(G)** Diagram depicting the curated (yellow background) or newly generated (blue background) datasets used in this study, along with the developed data analysis pipeline. The colors of the text boxes indicate the origin of the resource (*in situ, in vivo, in vitro*).

Abbreviations: ASPS, Alveolar soft part sarcoma; KTS, amino acid motif containing lysine-threonine-serine amino acids within WT1 CDS; BLK, B lymphocyte kinase; BAIAP3, BAI1-associated protein 3; (BAIAP3), BCOR, BCL-6 Corepressor; (BCOR), CIC, Capicua transcriptional repressor; CACNA2D2, Calcium Voltage-Gated Channel Auxiliary Subunit Alpha2delta 2; ChIP-seq, chromatin immunoprecipitation followed by sequencing; CCND1, Cyclin D1; DDLS, dedifferentiated liposarcoma; ENT4, Equilibrative nucleoside transporter-4; FGFR4, Fibroblast Growth Factor Receptor 4; EWSR1::WT1, fusion protein of EWSR1 and WT1 proteins; GIST, gastrointestinal stromal tumor; IRS, immune reactive score; IHC, immunohistochemistry; IQCG, IQ Motif Containing G gene; IGF2, Insulin-like Growth Factor 2; JAK3, Janus Kinase 3; JN-DSRCT-1shWT1, SK-DSRCT-2shWT1, JN-DSRCT-1 or SK-DSRCT-2 cell line models with shRNA-mediated KD of EWSR1::WT1; MFH, malignant fibrous histiocytoma; MPNST, malignant peripheral nerve sheath tumor; MeT-5AEWSR1::WT1, MeT-5A mesothelial cell line models with ectopic overexpression of either EWSR1::WT1 - KTS, EWSR1::WT1 + KTS, or EWSR1::WT1 +/- KTS; MYOD1, Myogenic Differentiation 1; NTRK3, Neurotrophic Receptor Tyrosine Kinase 3; PDGFA, Platelet-Derived Growth Factor Subunit A; RMS, rhabdomyosarcoma; RNA-seq, RNA-sequencing; shWT1, shRNA targeting EWSR1::WT1 mRNA; shRNA, short hairpin RNA; siRNA, small-interfering RNA; SRCS, small round cell sarcomas; siWT1, siRNAs targeting EWSR1::WT1 mRNA; SE, super enhancer; TMA, tissue microarray; UMAP, uniform manifold approximation and projection; WDLS, well differentiated liposarcoma.

**Supplementary Tables**

### Supplementary Table S1. DEP analysis of JN-DSRCT-1 and SK-DSRCT2 upon KD of EWSR1::WT1.

[Excel]

### Supplementary Table S2. ROSE super-enhancer analysis results for JN-DSRCT-1 and MeT-5A cell lines.

[Excel]

### Supplementary Table S3. *CACNA2D2* gene set.

[Excel]

### Supplementary Table S4. CACNA2D2 signature, EWSR1::WT1 signature, scCACNA2D2 signature and scEWSR1::WT1 signature.

[Excel]

### Supplementary Table S5. Solid tumor tissue collection overview.

[Excel]

### Supplementary Table S6. Oligonucleotide sequences used in this study.

[Excel]

### Supplementary Table S7. GEO series and accession codes for ChIP-seq data shown in Figure 1B and Supplementary Figure S2G.

[Excel]

### Supplementary Table S8. Accession codes for ChIP-seq datasets used in ROSE super-enhancer analysis.

[Excel]

### Supplementary Table S9. Single-cell RNA-seq data accession codes.

[Excel]

### Supplementary Table S10. Accession codes for all microarray datasets from normal and cancer tissue samples.

[Excel]
